# Sus1 Modulates Chromatin Remodeling and Gene Expression via the Cell Wall Integrity Pathway in *Saccharomyces cerevisiae*

**DOI:** 10.1096/fj.202504656RR

**Published:** 2026-04-28

**Authors:** Mónica Pavón‐Vergés, Raúl García, José M. Rodríguez‐Peña, Ana Belén Sanz, Javier Arroyo

**Affiliations:** ^1^ Departamento de Microbiología y Parasitología, Facultad de Farmacia Universidad Complutense de Madrid Madrid Spain

**Keywords:** chromatin remodeling, CWI pathway, gene expression, H2B deubiquitination, MAPK signaling, SAGA complex, Slt2, Sus1

## Abstract

Stressful situations that compromise the integrity of the fungal cell wall activate a transcriptional reprogramming mainly regulated by the MAPK Slt2 and the transcription factor Rlm1. Chromatin remodeling during this response involves the coordinated action of the SAGA complex, through its histone acetyltransferase Gcn5, and the SWI/SNF complex. Here, we define the specific contribution of another SAGA subunit, Sus1, which is also part of the TREX‐2 complex and the deubiquitinating module, in this process. Deleting *SUS1* has a widespread impact on the transcriptional program controlled by the cell wall integrity (CWI) pathway. During cell wall stress, Sus1 associates with CWI‐responsive genes as part of the SAGA complex through a mechanism involving Slt2, Rlm1, SWI/SNF, and SAGA itself. This association facilitates pre‐initiation complex assembly and RNA Pol II progression. Loss of Sus1 reduces histone H3 eviction and nucleosome displacement at CWI‐dependent genes under stress. Notably, this function is independent of histone H2B deubiquitination catalyzed by Ubp8. Moreover, the double *sus1*Δ *gcn5*Δ mutant exhibits additive effects on chromatin remodeling of CWI‐dependent genes under stress and on cell wall stress‐related phenotypes, indicating that Sus1 plays a direct role in this remodeling, acting via the CWI pathway independently of Gcn5‐mediated histone acetylation. These findings highlight a functional cooperation between Sus1 and Gcn5 in promoting transcriptional activation under stress and reveal new insights into the modular regulation of the SAGA complex during adaptive responses in fungi.

## Graphical abstract

Cell wall stress in yeast triggers transcriptional reprogramming through the CWI pathway. This study identifies Sus1, a subunit of SAGA and TREX‐2, as a key regulator in this process. Sus1 associates with stress‐responsive genes, promoting pre‐initiation complex assembly, chromatin remodeling, and RNA Pol II progression independently of H2B deubiquitination. The additive effects observed in double *sus1*Δ *gcn5*Δ mutants demonstrate that Sus1 functions independently of Gcn5, emphasizing their cooperative yet distinct roles in stress‐induced transcriptional activation.

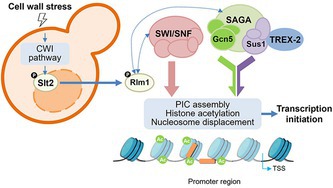

## 1 Introduction

Yeast cells utilize various mechanisms to adapt and survive under different stress conditions or external stimuli. Most of these mechanisms are regulated by MAPK signaling pathways, whose high degree of evolutionary conservation underscores their importance for proper cellular function [1].

The cell wall of *Saccharomyces cerevisiae* is an essential structure that surrounds the cell and protects it from adverse and changing environmental conditions [2]. Stressful situations that threaten the integrity of the fungal cell wall activate, among other responses, a transcriptional reprogramming primarily controlled by the CWI through the MAPK Slt2 [3, 4, 5]. In response to cell wall stress, Slt2 phosphorylation activates the transcription factors SBF and Rlm1, with Rlm1 primarily responsible for driving the transcriptional output of the CWI pathway [6, 7]. *RLM1* and *SLT2* are also upregulated in response to cell wall stress through an Rlm1‐dependent mechanism, creating a positive feedback loop essential for full CWI transcriptional activation [8]. In addition to its role in phosphorylating Rlm1, Slt2 also associates with DNA to interact with RNA polymerase II (Pol II), thereby moving along the coding region during transcriptional elongation [9].

During stressful conditions, gene expression must be precisely regulated to ensure appropriate levels of proteins in both quantity and timing. The complexity of DNA packaged into chromatin highlights the removal of nucleosomes at the promoter as a key step in transcriptional initiation [10]. In this context, the SWI/SNF ATP‐dependent chromatin remodeler complex is recruited to CWI‐dependent genes through direct interaction with Rlm1. The activity of SWI/SNF is critical for modifying chromatin organization at promoters and surrounding regions, enabling Rlm1 to access binding sites that are otherwise blocked by positioned nucleosomes and Pol II, thereby triggering transcription [11]. This process is supported by the action of the transcriptional coactivator Spt‐Ada‐Gcn5‐acetyltransferase (SAGA) complex. SAGA is a multisubunit complex (containing 18–20 proteins) with two known enzymatic modules that facilitate acetylation and deubiquitination of histones and non‐histone substrates (reviewed in [12, 13]). The acetylation module includes the H3 histone acetyltransferase (HAT) Gcn5 along with Ada2, Ada3, and Sgf29, while the deubiquitination module (DUBm) contains the H2B histone deubiquitinase (DUB) Ubp8, Sgf11, Sgf73, and Sus1. Under cell wall stress, the SAGA complex is recruited to the promoter of CWI‐responsive genes in a manner dependent on Slt2, Rlm1, and SWI/SNF. There, this complex, through its histone acetylase activity mediated by Gcn5, collaborates with the SWI/SNF complex to achieve the necessary nucleosome displacement and chromatin remodeling required for full gene expression via the CWI pathway [14].

Optimal transcription of specific genes depends on a timing cycle of H2B ubiquitination and deubiquitination [15]. Specifically, Ubp8, as part of the SAGA DUBm, reverses the ubiquitination of H2B at Lys 123 (H2BK123), which is catalyzed by Rad6/Bre1 [16, 17]. Removing ubiquitin from H2B helps regulate Lys4 H3 trimethylation at promoter regions, a marker linked to transcriptional activation [16, 17, 18, 19, 20]. Furthermore, H2B deubiquitination is crucial during elongation to recruit Ctk1, which phosphorylates Ser2 of the Pol II CTD [21]. This phosphorylation encourages the recruitment of the methyltransferase Set2, which methylates histone H3 Lys36 in the coding regions of actively transcribed genes, a process necessary for subsequent steps in transcription elongation through direct interaction with Pol II [22, 23].

Gene expression is regulated not only at the transcriptional level. In eukaryotic cells, the nuclear membrane separates transcription from translation, making mRNA transport between the nucleus and cytoplasm through the nuclear pore complex (NPC) a crucial step in gene expression regulation. Sus1, a small 11 kDa protein highly conserved in eukaryotes, enhances gene expression by coupling and coordinating transcription with the nucleocytoplasmic transport of mRNA in both time and space [24]. Sus1 is a component of DUBm of the SAGA complex and the nuclear pore‐associated TREX‐2 (Transcription export 2) complex [24]. TREX‐2 (Sac3‐Thp1‐Cdc31‐Sus1) is an mRNA export complex that binds to various nucleoporins at the nuclear pore basket and, together with the export receptor Mex67‐Mtr2, promotes the nuclear export of mRNPs [25]. Sac3 acts as a scaffold protein, dividing the complex into three modules [26, 27, 28]: its N‐terminal region binds to the main mRNA nuclear export factor Mex67‐Mtr2; its middle region forms a complex with Thp1 and Sem1, creating a nucleic acid‐binding module that also interacts with the Mediator Complex; and its C‐terminal region interacts with Cdc31 and two copies of Sus1, mediating the binding of TREX‐2 with Nup1 and Nup60 basket nucleoporins from the NPC. This facilitates the localization of genes such as *GAL1* [29, 30]. Mutations that disrupt Sac3's binding with Sus1, Cdc31, or Thp1 lead to problems in mRNA export and cell growth [26, 30].

In yeast, Sus1 functions as a transcriptional regulator that controls the transcription of 9% of genes [24]. Under transcriptionally active conditions, Sus1 is recruited to the promoters of the SAGA‐dependent *GAL1*, *ADH1*, and *PHO84* genes as part of the SAGA complex to promote PIC assembly independently of H2B deubiquitination [24, 31]. Additionally, Sus1 interacts with the elongating form of Pol II, phosphorylated on CTD Ser5 and Ser2, facilitating its association with the *ARG1* gene coding sequence in a manner dependent on SAGA and TREX‐2 [32]. It also engages with the mRNA export factors Yra1 and Mex67 to ensure efficient elongation and link transcription to export [32]. Indeed, the tethering of the *GAL1* gene to the nuclear periphery relies on Sus1, which enhances gene expression efficiency by coupling transcription with mRNA export [33, 34]. The functions of Sus1, while central, are not limited to the nucleus but extend to the cytoplasm, where it appears to be functionally involved in mRNA metabolism by interacting with the degradation machinery at P‐bodies and stress granules [35].

Despite extensive information on Sus1, studies examining its role in stress responses are limited [36, 37, 38]. Here, we report on the role of Sus1 in regulating the transcriptional response of *S. cerevisiae* under stress conditions that disturb cell wall structure. To achieve this, we characterized the impact of Sus1 on the global transcriptional response of cells to Congo red (CR) and its contribution to chromatin remodeling and to the recruitment of the transcriptional machinery to genes induced under CWI‐activating conditions. Sus1 promotes PIC assembly and chromatin remodeling at CWI‐dependent genes in response to cell wall stress, independent of Ubp8‐catalyzed histone H2B deubiquitination. Additionally, our results demonstrate not only a direct role for Sus1 in chromatin remodeling under these conditions but also a cooperative effect with Gcn5.

## 2 Materials and Methods

### 2.1 Yeast Strains, Plasmids, and Growth Conditions

The yeast strains used in this study, along with their genotypes and sources, are listed in Table S1. The *sus1*Δ mutant was obtained by replacing the *SUS1* gene in the BY4741 strain with the *KanMX4* marker using the SFH PCR‐based method described by Wach et al. [39]. The double mutant *sus1*Δ *gcn*5Δ (which is the same as BY4741 but with *sus1::KanMX4* and *gcn5::KanMX4*) was generated through sporulation and tetrad analyses using standard yeast genetics techniques after creating the corresponding diploid heterozygous disruptant. The tagged strain WT‐*SUS1*‐Myc, along with the corresponding mutants, was obtained using a one‐step PCR‐mediated gene modification technique as described by Longtine et al. [40]. The fragment containing 13xMyc‐His3 was amplified by PCR using the pFA6a‐13Myc‐His3MX6 plasmid as the template, with primers as outlined by Longtine et al. [40]. This fragment was integrated via homologous recombination into the *SUS1* locus, and correct integration was confirmed with a PCR‐based strategy. Expression of the Myc‐tagged protein was further verified by western blotting, showing a band corresponding to the expected molecular weight of approximately 40 kDa. The same approach was employed to construct the WT‐*SAC3‐*Myc strain. The strains *sus1*Δ‐*RLM1*‐HA, *sus1*Δ‐*UBP8*‐HA, *sus1*Δ‐*SPT20*‐Myc, and *ubp8*Δ (pTDH3‐3xHA‐Ub) were generated by replacing the *SUS1* or *UBP8* genes in the respective WT strain with the *KanMX4* or *HIS3* marker as described by Wach et al. [39]. For the creation of the *sac3*Δ‐*RLM1*‐Myc and *ubp8*Δ‐*RLM1*‐HA strains, the plasmids pFA6a‐13Myc‐His3MX6 and pFA6a‐3HA‐His3MX6 described by Longtine et al. [40] were used. The fragments containing 13xMyc‐His3 or 3xHA‐His3 were amplified by PCR using the oligonucleotides including sequences specific to the *RLM1* gene. All oligonucleotides used are listed in Table S2. The plasmid YEp181‐*RLM1*‐6xMyc, which expresses the fusion protein Rlm1‐6xMyc under the control of the native *RLM1* promoter, was constructed by replacing the 234 bp *Nde*I/*Sac*I fragment from the YEp181‐*RLM1* plasmid [41] with the 500 bp *Nde*I/*Sac*I fragment from the pRS305Rlm1 plasmid [42], which includes the C‐terminal sequence of *RLM1* fused to 6xMyc.

Yeast cells were routinely cultured overnight in yeast extract peptone dextrose (YPD) medium (1% yeast extract, 2% peptone, and 2% glucose) or synthetic‐defined (SD) medium (0.17% yeast nitrogen base, 0.5% ammonium sulfate, and 2% glucose), supplemented with the necessary amino acids for cells carrying plasmids. The cultures were incubated at 220 rpm and 24°C until they reached an optical density of 0.8–1 (A600). Then, the cultures were diluted to 0.2 (A600) in YPD medium and incubated for an additional 2 h. At this stage, the cultures were divided into two parts: one part continued to grow under the same conditions (non‐treated culture), while the other was supplemented with CR at a concentration of 30 μg/mL (Merck KGaA, Darmstadt, Germany). Cells were collected at the specified time points and processed according to the experimental plan.

### 2.2 Phenotypic Analyses

Cells were cultured overnight at 24°C in a YPD medium, and exponentially growing cultures were adjusted to an optical density of 0.2 (A_600_) (∼3 × 10^3^ cells per μL). 1:5 serial dilutions of yeast cultures were spotted using a 48‐pin Multi‐Blot replicator (407AH model, V&P Scientific) onto YPD plates and YPD plates supplemented with 50 μg/mL of CR. The plates were incubated at 30°C, and growth was monitored after 48 h.

### 2.3 Western Blotting Assays

The procedures used for immunoblotting analyses, including cell collection and lysis, protein extraction, SDS–PAGE fractionation, and transfer to nitrocellulose membranes, were performed as previously described [43] using the Odyssey Infrared Imaging System (LI‐COR Biosciences, Lincoln, NE, USA). The detection of Slt2 was accomplished using the monoclonal antibody anti‐Mpk1 (Clone E‐9, sc‐1333189, Santa Cruz Biotechnology, Dallas, TX, USA), and the phosphorylated version was detected using the polyclonal antibody anti‐phospho‐p44/42 MAPK (Thr202/Tyr204) (4370, Cell Signaling Technology, Danvers, MA, USA). The detection of HA and Myc was performed using monoclonal antibodies anti‐HA (Clone C12CA5, 11666606001, Roche Diagnostics GmbH, Mannheim, Germany) and anti‐Myc (Clone 4A6, 05‐724, Merck KGaA, Darnstadt, Germany), respectively. To monitor protein loading, Glucose‐6‐phosphate dehydrogenase levels were determined using an anti‐G6PDH polyclonal antibody (A9521, Sigma–Aldrich, St. Louis, MO, USA). The secondary antibodies used were IRDye 800CW goat anti‐rabbit (926‐32211) and IRDye 680LT goat anti‐mouse (926‐68020), both from LI‐COR Biosciences. All antibodies were used at the dilutions recommended by the manufacturers. Protein band quantification was carried out by densitometric analysis using Image Studio Lite software (version 5.0) from LI‐COR Biosciences.

### 2.4 Microarray Experiments

Total RNA isolation and purification were conducted as described elsewhere [44]. Genome‐wide transcriptional profiles were obtained using the Affymetrix GeneChip Yeast Genome 2.0 arrays (Affymetrix, Santa Clara, CA, USA). cDNA synthesis, chip hybridization, image analysis, data processing, and statistical analyses were performed as previously outlined [45]. The scanned files (.CEL) were converted into gene expression signals using the RMA algorithm included in the Affymetrix Expression Console. For each experimental condition, three microarray experiments, corresponding to three independent biological samples, were processed and analyzed. Fold changes between experimental conditions were calculated as the ratio of the mean gene expression signal. Genes were classified as upregulated or downregulated when their expression ratio under the tested conditions was ≥ 2 or ≤ 0.5, respectively. Statistical analysis was performed using the Student's *t*‐test, with a significance level set at *p* < 0.05. Genes exhibiting statistical significance were selected for further analysis.

To assess the impact of the *sus1*Δ mutation on gene induction following CR treatment (30 μg/mL, 3 h) in the WT strain, we compared the response of the mutant (mutant ratio, *sus1*Δ CR/*sus1*Δ) with the response of the WT strain (WT ratio, WT CR/WT). We defined a significant reduction in gene induction as a mutant ratio/WT ratio below the threshold of 0.65, as described by García et al. [46]. Additionally, genes with a mutant ratio below 1.65 were considered not upregulated. The same threshold for the relationship between the *sus1*Δ/WT ratio versus WT CR/WT ratio was used to determine genes whose basal expression levels depended on *SUS1*. Clustering analysis was conducted using the MeV MultiExperiment Viewer, version 4.9 (The Institute for Genomic Research, Rockville, MD, USA). The microarray data described here follow the Minimum Information About a Microarray Experiment (MIAME) recommendations.

### 2.5 Reverse Transcription Quantitative PCR (RT‐qPCR)

Total RNA isolation and purification were performed as detailed elsewhere [44]. RT‐qPCR assays were conducted according to the established protocol described in the same study. To quantify gene expression levels, the abundance of each gene was normalized to the *ACT1* reference transcript for cDNA input normalization, as the Ct values for this gene across three independent biological replicates remained largely unchanged between non‐treated (17.63 ± 0.17) and CR‐treated (17.31 ± 0.29) samples. The relative gene expression levels between the tested conditions were calculated using the 2^(−ΔΔCt)^ method [47]. Primer sequences are provided in Table S2.

### 2.6 Flow Cytometry

Flow cytometry assays were conducted to quantify Kdx1 expression levels in WT and mutant cells. To achieve this, the strains were individually transformed with the *KDX1*‐GFP reporter plasmid, which includes both the promoter region and coding regions of *KDX1* fused to *GFP* [48]. Transformants derived from each mutant strain were grown overnight in SC‐Ura medium at 24°C and subsequently processed as described above. A 100 μL aliquot of the culture was harvested by centrifugation, washed with PBS, and resuspended in 500 μL of PBS containing 4% formaldehyde for 15 min at 4°C. The cell pellet was rewashed with PBS and analyzed using a Guava easyCyte flow cytometer (Merck Millipore, Darmstadt, Germany) equipped with a 488‐nm blue laser. Green fluorescence emission was captured using a 525/30 filter. The mean fluorescence intensity (MFI) and the percentage of GFP‐positive cells were determined for each sample with InCyte software (Merck Millipore, Darmstadt, Germany).

### 2.7 Chromatin Immunoprecipitation Assays

ChIP was performed as described in previous studies [11, 49]. The antibodies utilized in these experiments included monoclonal anti‐Myc (clone 9E10, sc‐40 X, Santa Cruz Biotechnology), polyclonal anti‐HA (ab9110, Abcam, Cambridge, UK), monoclonal anti‐Rpb1 (clone 8WG16, 664 906, BioLegend, San Diego, CA, USA), a polyclonal anti‐Snf2 antibody generously provided by Joseph C. Reese (Pennsylvania State University, USA), polyclonal anti‐H2B (39 237, Active Motif, Carlsbad, CA, USA), and polyclonal anti‐H3 (ab1791, Abcam, Cambridge, UK).

The immunoprecipitated DNA was quantified by qPCR using primers that amplified the following regions (locations are indicated by the distance from the respective ATG initiation codon): *KDX1*‐BOX1 (Rlm1 binding site at the promoter), −453/−313; *KDX1*‐POL II (promoter/ORF), −143/+56; *KDX1*‐ORF2 (ORF), +555/+687; *KDX1*‐ORF3 (3′end ORF), +1210/+1359; *YPL088W*‐BOX (Rlm1 binding site at the promoter), −573/−423; *YPL088W*‐POL II (promoter/ORF), −91/+55; *YPL088W*‐ORF2 (ORF), +562/+712; *YPL088W*‐ORF3 (3′end ORF), +911/+1064; *PRM5*‐BOX (Rlm1 binding site at the promoter), −287/−106; *PRM5*‐POL II (promoter/ORF), −106/+41; *PRM5*‐ORF2 (ORF), +393/+545; *PRM5*‐ORF3 (3′end ORF), +805/+951. Primer sequences are listed in Table S2.

The fold enrichment (FE) at specific DNA regions was calculated using the Comparative Ct Method [50]. An intergenic region of *ChroV*, which does not exhibit variation in expression under cell wall stress, served as a control. The Ct value of the input sample was subtracted from the Ct value of the immunoprecipitated sample to determine ΔCt for both the control sequence (ΔCt_cont_) and the target DNA (ΔCt_exp_) under each condition. Finally, FE was calculated using the formula: FE = 2^−[ΔCtexp−ΔCtcont]^. The data represent the mean ± standard deviation of at least three independent experiments.

### 2.8 Nucleosome‐Scanning Analysis

MNase digestion of chromatin, protein degradation, and DNA purification were performed according to the protocol described by Liu et al. [51] with some modifications. Initial titrations were conducted to determine the optimal amount of MNase required to yield more than 80% mononucleosomal DNA. Ultimately, 50 and 15 U of micrococcal nuclease (Worthington Biochemical, Lakewood, NJ, USA) were used for the WT and *sus1*Δ strains, respectively. Control samples of genomic DNA were obtained as described by Sekinger et al. [52], using varying amounts of MNase (0, 1.2, 1.4, and 1.6 U). Genomic DNA samples were purified using Qiagen columns (Qiagen, Hilden, Germany). Electrophoresis on a 1.5% agarose gel was conducted to separate the genomic DNA and MNase‐treated chromatin, followed by excision and purification of mononucleosome‐sized fragments (140‐220 bp). The purified DNA samples were analyzed by qPCR using a set of 11 overlapping primer pairs listed in Table S2. Each pair generated PCR products of approximately 100 ± 8 bp, with an overlap of approximately 30 ± 10 bp within the *KDX1* (−699 to +161 bp) promoter region. The nucleosomal DNA enrichment level of a specific DNA region was determined by calculating the ratio of the amount of PCR product obtained from the experimental samples to that of the genomic DNA (control samples).

### 2.9 Statistical Analysis

Data are expressed as mean ± standard deviation (SD). Comparisons between two groups were performed using Student's *t*‐test, while comparisons among multiple groups were conducted using one‐way analysis of variance (ANOVA). Data normality was assessed before statistical analysis using the Shapiro–Wilk test, confirming that the datasets were normally distributed (*p* > 0.05). GraphPad Prism 8.0 software was used for data analysis. Significant difference thresholds were considered at *p < 0.05, **p < 0.01, ***p < 0.001, and ****p < 0.0001.

## 3 Results

### 3.1 Subunits of the SAGA DUBm and TREX2 complex Are Required for the Transcriptional Response Triggered by Cell Wall Stress Through the CWI Pathway

In a previous extensive screen conducted in our laboratory [11], we identified 159 genes required to induce the cell wall stress‐responsive gene *KDX1/MLP1*. In addition to components of the SWI/SNF complex and SAGA HATm, whose roles in CWI transcriptional responses have already been characterized [11, 14], subunits of the SAGA DUBm (Sgf73) and TREX‐2 (Sem1) were also discovered, indicating a possible connection of these complexes with the CWI pathway.

**FIGURE 1:**
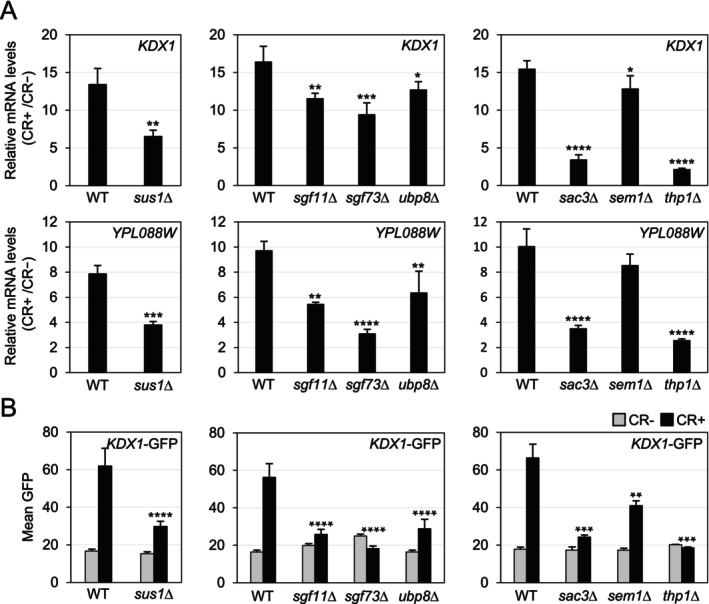
The SAGA (DUBm) and TREX‐2 complexes are essential for the transcriptional activation of CWI‐responsive genes. (A) mRNA levels of *KDX1* and *YPL088W* genes were measured by RT‐qPCR in the WT strain and the indicated mutants after 3 h of CR (30 μg/mL) treatment. Expression values represent the ratio of CR‐treated to untreated cells. The data represent the mean and standard deviation from at least three independent experiments. (B) *KDX1* transcriptional activation was evaluated by flow cytometry in the same strains as in (A), transformed with the *KDX1*‐GFP reporter plasmid, both with and without CR (3 h, 30 μg/mL). The data represent the mean fluorescence intensity and standard deviation of at least three independent transformants. Statistical significance was assessed using a one‐way analysis of variance (ANOVA) or Student's *t*‐test, comparing mutants to the WT strain. Only significant differences are shown: *p < 0.05, **p < 0.01, ***p < 0.001, and ****p < 0.0001.

To determine the role of these complexes in the cell wall stress transcriptional response, we evaluated how deleting the subunits of both complexes (SAGA DUBm: Sus1, Sgf11, Sgf73, and Ubp8; and TREX‐2: Sus1, Sac3, Sem1, and Thp1) affected the mRNA levels of two CWI‐dependent genes (*KDX1* and *YPL088W*) under cell wall stress mediated by CR. CR, like Calcofluor white, induces cell wall stress by binding to chitin and disrupting cell wall assembly [7, 53]. As shown in Figure 1, the induction of *KDX1* and *YPL088W* mRNA levels by CR was reduced when Sus1 was absent. Furthermore, transcript levels were altered in all SAGA DUBm and TREX‐2 mutants analyzed, notably in *sgf73*Δ, *sac3*Δ, and *thp1*Δ strains. Flow cytometry analysis of Kdx1 protein levels in these mutants, transformed with a *KDX1*‐GFP fusion construct, and grown with or without CR, also demonstrated that all subunits significantly affected Kdx1 expression (Figure 1B). To rule out the possibility that the blockade of transcriptional activation was related to defects in signaling through the CWI pathway, we characterized the activation of the MAPK Slt2. As shown in Figure S1, the phosphorylation levels of Slt2 induced by CR were not significantly altered in any of the mutants compared to the wild‐type (WT) strain. Therefore, signaling through the CWI pathway remains functional in the absence of the SAGA DUBm or TREX‐2 complex.

### 3.2 The Deletion of *SUS1* Has a Global Impact on the CWI Transcriptional Program

**FIGURE 2:**
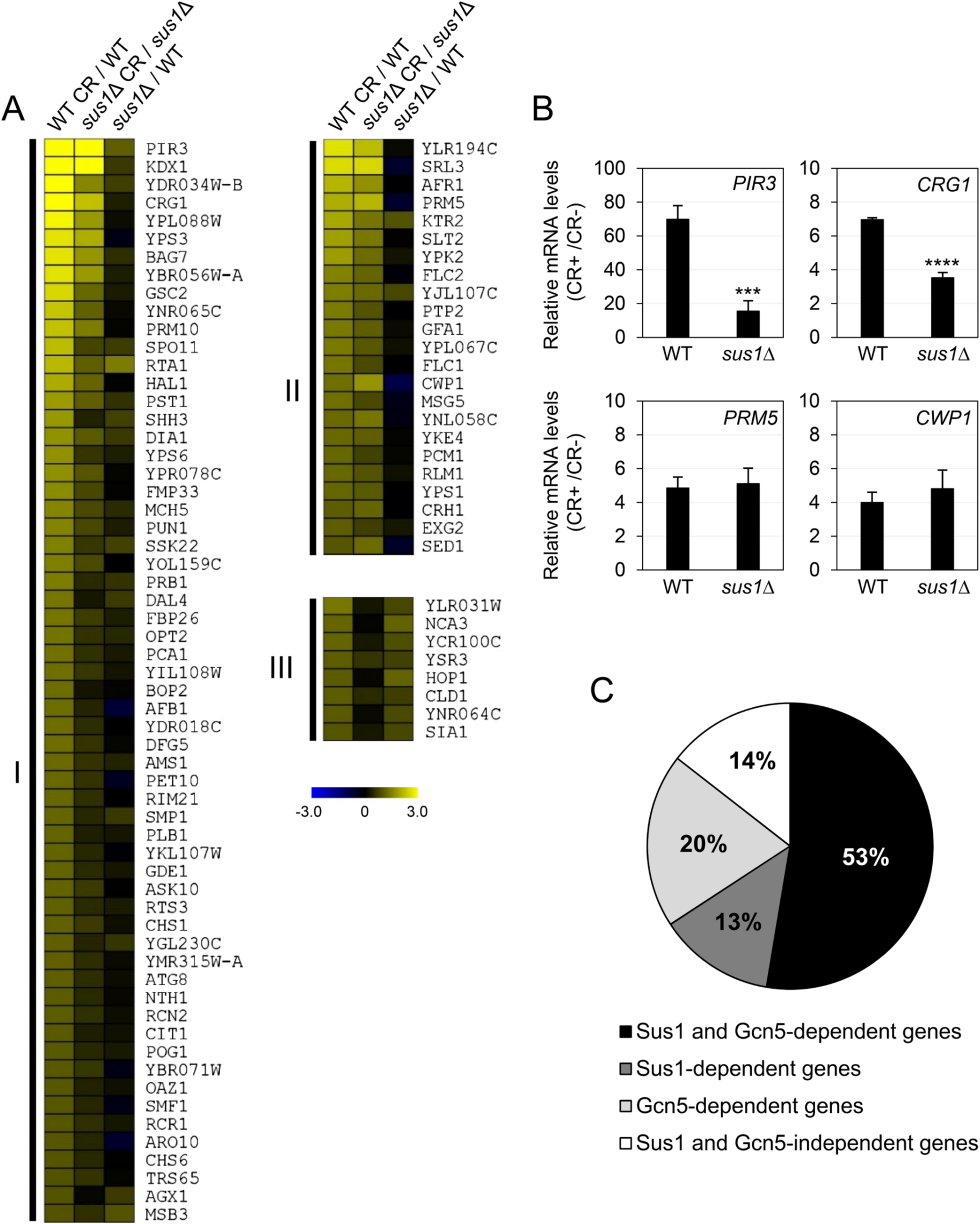
Genome‐wide expression profiles of WT and *sus1*Δ strains after CR treatment. (A) The heat map generated using MeV 4.9 software illustrates gene expression ratios that compare the transcriptional response to CR (3 h, 30 μg/mL) (treated vs. untreated) in WT and *sus1*Δ strains (two columns on the left). The gene expression ratio of the *sus1*Δ mutant relative to the WT strain under non‐stress conditions is shown in the third column. The analysis includes genes upregulated in the WT strain upon CR treatment grouped into clusters I to III based on their dependence on Sus1 for baseline expression and CR activation (refer to *Materials and Methods* for details). The color saturation reflects the log_2_ expression ratio, as indicated by the scale bar. (B) mRNA levels of selected CWI‐responsive genes, categorized as Sus1‐dependent (*PIR3* and *CRG1*) or Sus1‐independent (*PRM5* and *CWP1*), were analyzed by RT‐qPCR in WT and *sus1*Δ strains after CR (3 h, 30 μg/mL) treatment. Values represent the ratio between CR‐treated and untreated cells. Data are presented as the mean and standard deviation from at least three independent experiments. Results were analyzed using Student's t‐test,comparing mutant and WT samples. Only significant differences are indicated: ***p < 0.001, and ****p < 0.0001. (C) Schematic comparison of the transcriptional profiles of *sus1*Δ and *gcn5*Δ [14] mutants after CR treatment relative to the WT strain showing data on the percentages of genes dependent on Sus1, Gcn5, or both.

To investigate the role of Sus1, the component shared by both complexes SAGA DUBm and TREX‐2, in the CWI transcriptional program, we examined the transcriptomes of WT and *sus1*Δ strains under normal and cell wall stress conditions (CR, 3 h). Microarray analysis showed that 91 genes were induced (transcript‐levels ratio ≥ 2) and 13 genes were repressed (transcript levels ratio ≤ 0.5) following CR treatment in the WT strain. These findings align with our previously published results [7, 11, 14, 46]. Conversely, only 34 genes were induced in the *sus1*Δ mutant, with no genes repressed (Figure 2). A comprehensive list of genes involved in the response, along with their expression ratios, is available in Table S3. A detailed comparison of the transcriptional induction profiles of WT and mutant cells (see Section 2) identified three gene clusters related to the stress response and Sus1 dependence: (1) genes whose induction depended on Sus1 (60 genes; 66%); (2) genes induced by cell wall stress independently of Sus1 (23 genes; 25%); and (3) a small group of eight genes (9%) with basal expression levels that relied on Sus1 (Figure 2A; Table S3). Therefore, Sus1 was responsible for activating nearly 70% of CWI‐regulated genes. The transcriptional activation of these genes, including both Sus1‐dependent (*PIR3* and *CRG1*) and independent (*PRM5* and *CWP1*) genes, was further validated through reverse transcription quantitative PCR (RT‐qPCR) (Figure 2B). Additionally, to study the similarities of this response with those related to the HAT module of the SAGA complex, we compared the *sus1*Δ transcriptional profiles with those of a *gcn5*Δ mutant upon CR stress [14]. Results showed that over half of the CR‐induced transcriptional response depended on both Sus1 and Gcn5 (Figure 2C; Table S3), further supporting a cooperative role of the SAGA modules in regulating gene expression responses via the CWI pathway.

To determine if Sus1 is also necessary for the CWI transcriptional response triggered by other chemical agents that cause cell wall stress through different mechanisms, we examined the expression levels of *KDX1* and *PIR3* in WT and *sus1*Δ cells grown in the presence of zymolyase, which modifies the cell wall via its β‐1,3‐glucanase, protease, and chitinase activities [54] or caspofungin, which inhibits β‐1,3‐glucan synthase [55]. As shown in Figure S2, deleting *SUS1* also reduces the expression of these genes under these stress conditions.

### 3.3 Cell Wall Stress Triggers the Recruitment of Sus1 to Sus1‐Dependent Genes Through a Mechanism Involving Slt2, Rlm1, SWI/SNF, and SAGA

**FIGURE 3:**
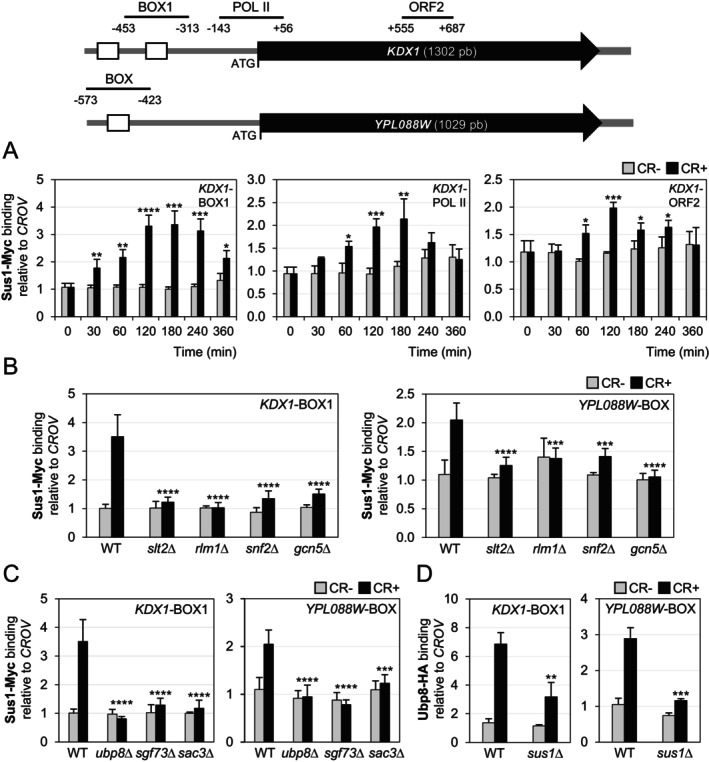
Sus1 associates with CWI‐related genes in an Slt2‐, Rlm1‐, SAGA‐, and SWI/SNF‐dependent manner as part of the SAGA DUBm. A schematic diagram of the *KDX1* and *YPL088W* genes is depicted in the top panel, highlighting Rlm1‐binding sites with white boxes. The regions amplified by primers for ChIP experiments are shown as horizontal lines with their respective positions relative to the ATG start codon. (A) Sus1 binding to the *KDX1* gene was examined by ChIP in a WT strain expressing an integrated, epitope‐tagged Sus1‐Myc protein during CR treatment (30 μg/mL). The specific regions of *KDX1* analyzed are noted in the upper right corner. (B) Recruitment of Sus1 to the *KDX1* and *YPL088W* promoters (*KDX1*‐BOX1 and *YPL088W*‐BOX regions, respectively) was evaluated by ChIP in WT, *slt2*Δ, *rlm1*Δ, *snf2*Δ, and *gcn5*Δ strains expressing Sus1‐Myc, in the absence and presence of CR (3 h, 30 μg/mL). (C) Sus1 binding was assessed as in (B) in WT, *ubp8*Δ, *sgf73*Δ, and *sac3*Δ strains expressing Sus1‐Myc. (D) Ubp8 binding was assessed by ChIP in WT and *sus1*Δ strains expressing an integrated, HA‐tagged Ubp8 protein at the *KDX1* and *YPL088W* promoters (BOX1 and BOX regions, respectively). Data represent the mean and standard deviation of at least three independent experiments. Statistical significance was determined using Student's *t*‐test to compare treated (CR+) and untreated (CR‐) samples in (A), and either ANOVA or Student's *t*‐test to compare WT and mutant strains in the remaining panels. Only significant differences are indicated: *p < 0.05, **p < 0.01, ***p < 0.001, and ****p < 0.0001.

To understand how Sus1 regulates the CWI‐mediated transcriptional response, we first examined whether this protein is recruited in vivo to the *KDX1* gene in response to cell wall stress, using chromatin immunoprecipitation (ChIP). As shown in Figure 3A, Sus1 was not bound to any of the regions analyzed without stress. However, CR caused Sus1 to be noticeably recruited after 30 min of treatment to two regions of the *KDX1* promoter: the BOX1 region, which contains an Rlm1 binding site, and the POL II region that includes the ATG. This recruitment increased progressively, reaching a maximum after 2–3 h of treatment, with kinetics similar to those of Rlm1, Slt2, and other PIC components recruited to CWI‐dependent genes under cell wall stress, matching their gene expression profiles [11]. Additionally, as shown in Figure 3A, although to a lesser extent, Sus1 was also significantly recruited to the *KDX1* coding region. These results suggest that Sus1 plays a key role in transcription initiation under cell wall stress and also in elongation.

Sus1 binding, a result of CR stress, was completely abolished in the absence of Slt2 and Rlm1 (Figure 3B), indicating that a functional CWI pathway is necessary to direct Sus1 to its target genes. To better understand how Sus1 associates with chromatin, we also examined whether SWI/SNF and SAGA, which are responsible for restructuring the chromatin environment at the promoter level of CWI‐dependent genes [11, 14], are required for Sus1 recruitment to CWI‐gene promoters. As shown in Figure 3B, Sus1 binding to *KDX1* and *YPL088W* promoters was severely impaired under stress when either of the two catalytic subunits of SWI/SNF and SAGA HATm complexes, Snf2 and Gcn5, were absent. Therefore, targeting Sus1 to CWI‐dependent gene promoters, following activation of the CWI pathway, does not occur independently but requires the recruitment of Rlm1 and the coactivators SAGA and SWI/SNF.

Since Sus1 is part of two different complexes, SAGA and TREX‐2, we wondered how each complex contributes to Sus1's interaction with chromatin. To determine whether Sus1 binds to the *KDX1* gene as part of SAGA, we analyzed its binding by ChIP in the absence of Ubp8. In the *ubp8*Δ strain, Sus1 remains associated with the subunits of the TREX‐2 mRNA export complex; however, the association between SAGA and Sus1 is disrupted [56]. As shown in Figure 3C, Sus1 binding to CWI promoter genes (*KDX1* and *YPL088W*) under stress depends entirely on Ubp8. Therefore, Ubp8 and SAGA are necessary for targeting Sus1 to CWI‐dependent gene promoters. In contrast to the absence of Ubp8, removing Sgf73 impairs Sus1's association with SAGA and reduces Sus1 binding to Sac3 (the TREX‐2 complex) [32]. As expected, Sus1 recruitment to *KDX1* and *YPL088W* was completely abolished in the absence of Sgf73 (Figure 3C). To further investigate the potential role of the TREX‐2 complex, we examined Sus1 binding in a *sac3*Δ mutant under CR treatment. The absence of Sac3 significantly disrupts Sus1's association with TREX‐2, while Sus1's connection with SAGA remains mostly unchanged [32]. ChIP experiments with a *sac3*Δ strain showed that Sac3, like Ubp8, is also required for recruiting Sus1 to the promoters of the two analyzed genes (Figure 3C). However, while there were no significant differences in the Rlm1 levels in the absence of *SUS1* or *UBP8* compared to the WT, both with and without stress (Figure S3A,B), these levels were found to be severely compromised in the *sac3*Δ strain and were not restored by overexpressing Rlm1 from a multicopy plasmid (Figure S3B), suggesting that *RLM1* mRNAs, which were not affected in the *sac3*Δ strain (Figure S3C), are not exported in this mutant. Therefore, the dependence on TREX‐2 for the recruitment of Sus1 in response to stress may not be direct but rather an indirect effect caused by altered Rlm1 levels in the *sac3*Δ strain.

Additionally, we examined the in vivo interaction of Ubp8 and Sac3 with the same gene regions, indicating SAGA and TREX‐2 binding, using the corresponding epitope‐tagged strains. As shown in Figure 3D, Ubp8 associates with the *KDX1* and *YPL088W* promoters under stress conditions in a Sus1‐dependent manner. However, no association of Sac3 was observed with either of the two genes analyzed (Figure S4), further supporting the idea that under cell wall stress, Sus1 is recruited to the promoters of Sus1‐dependent genes *KDX1* and *YPL088W* as part of the SAGA complex.

### 3.4 Sus1 Promotes PIC Formation at CWI‐Regulated Genes Without Relying on Histone H2B Deubiquitination

**FIGURE 4:**
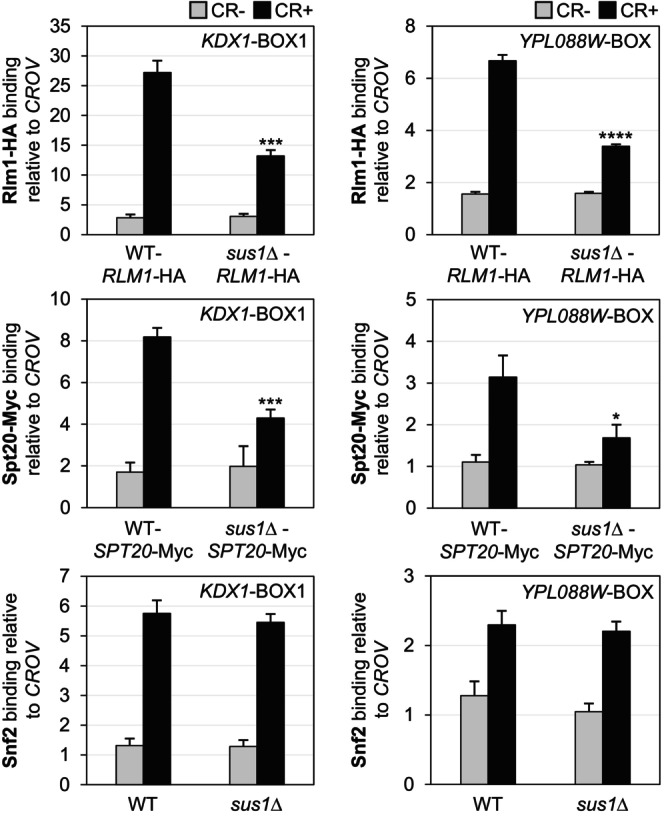
Effect of *SUS1* deletion on the recruitment of Rlm1, SWI/SNF, and SAGA complexes. The binding of Rlm1‐HA, Spt20‐Myc, and Snf2 to the *KDX1* and *YPL088W* promoters was analyzed by ChIP in the indicated strains after CR treatment (3 h, 30 μg/mL). The regions analyzed are shown in the upper right corner. Data represent the mean and standard deviation of at least three independent experiments. Results were analyzed using Student's *t*‐test, comparing mutant‐treated (CR+) and WT‐treated (CR+) samples. Only significant differences are indicated: *p < 0.05, ***p < 0.001, and ****p < 0.0001.

**FIGURE 5:**
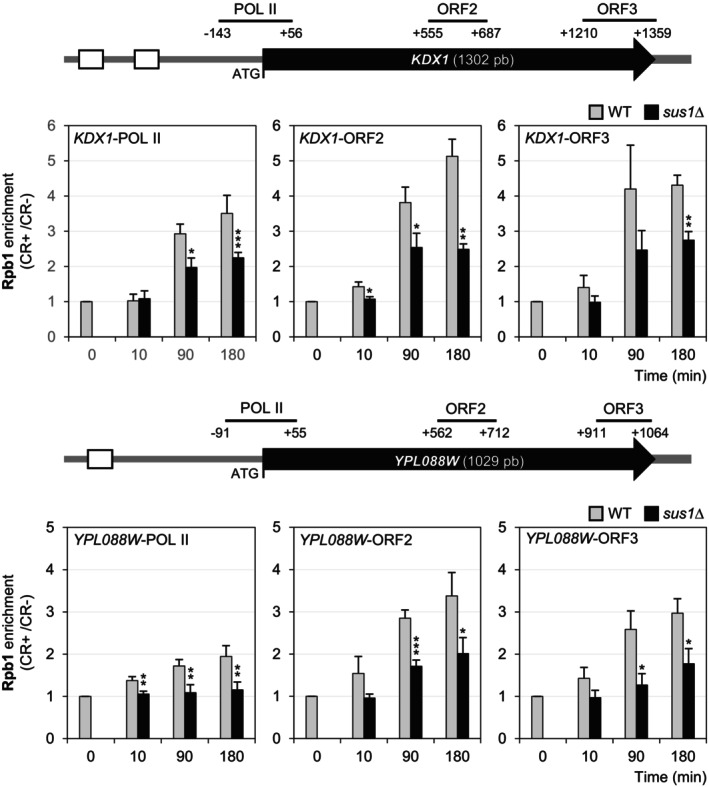
Deletion of *SUS1* alters Pol II progression at *KDX1* and *YPL088W* genes. The recruitment of the Rpb1 subunit of Pol II to the promoter (POL II region) and ORF (ORF2 and ORF3 regions) of *KDX1* (top panel) and *YPL088W* (bottom panel) was analyzed by ChIP in WT and *sus1*Δ strains after CR treatment (30 μg/mL) at the indicated time points. The regions analyzed are shown as horizontal lines in the schematic depiction of each gene, with their respective positions relative to the ATG start codon marked. Values indicate the ratio of binding between CR‐treated and untreated cells. Data reflect the mean and standard deviation of at least three independent experiments. Student's *t*‐test was employed to assess significant differences between WT and mutant samples (*p < 0.05, **p < 0.01, ***p < 0.001).

To elucidate the role of Sus1 in transcription initiation in response to cell wall stress, we investigated whether Sus1 participates in the formation of the PIC at *KDX1* and *YPL088W* promoters. We performed ChIP experiments to evaluate the recruitment of the transcription factor Rlm1 and the coactivators SWI/SNF and SAGA, both in the presence and absence of Sus1. As shown in Figure 4, the binding of Rlm1 and SAGA (Spt20) to these gene promoters significantly decreased after 3 h of CR treatment when Sus1 was absent. In contrast, and consistent with previous results for the *gcn5*Δ mutant [14], Snf2 binding remained unchanged in the *sus1*Δ mutant. These findings indicate that Sus1 is at least partly necessary for recruiting Rlm1 and SAGA to chromatin and that Sus1 enhances PIC assembly during the CR response, although it is dispensable for SWI/SNF recruitment. Next, we investigated whether Pol II binding to the *KDX1* and *YPL088W* genes was affected by the absence of Sus1. Chromatin from wild‐type and *sus1*Δ strains at various times during CR treatment was immunoprecipitated using an anti‐RNA Pol II Rpb1 antibody and analyzed across a promoter region, including the ATG and two coding regions (ORF2 and ORF3) of both genes. As shown in Figure 5, in both cases Pol II recruitment, measured as a ratio with and without CR, decreased by 40%–50% in the absence of Sus1. Therefore, Sus1 may play a role in Pol II's binding to DNA and its progression along the coding regions of these genes, which is promoted by CWI pathway activation.

**FIGURE 6:**
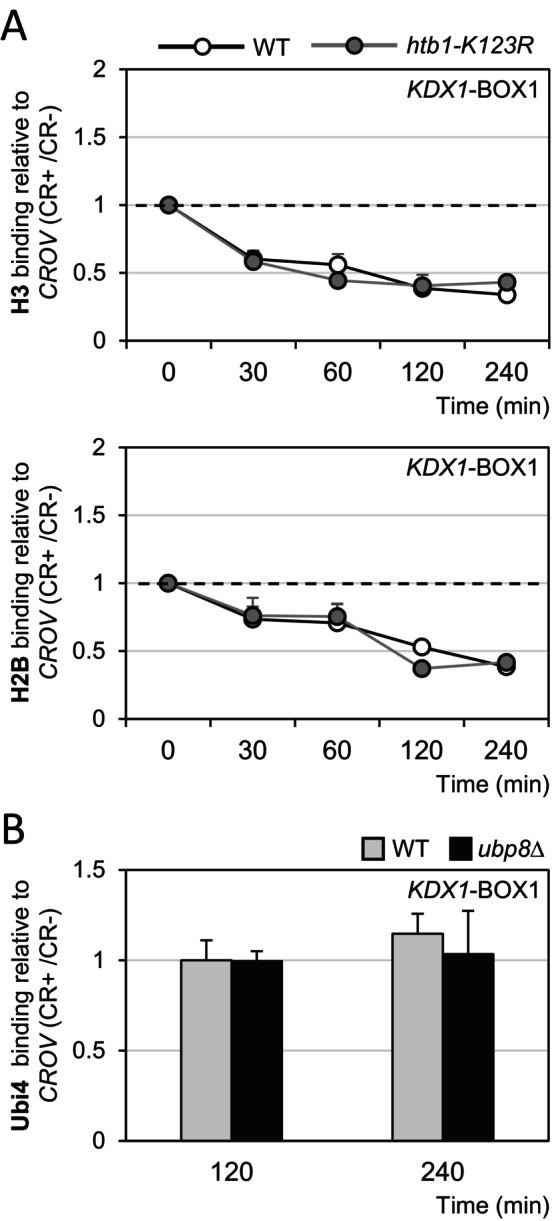
Chromatin remodeling at the *KDX1* promoter does not depend on ubiquitination/deubiquitination events. (A) The binding of histone H3 (top panel) and H2B (bottom panel) to the *KDX1*‐BOX1 promoter region was analyzed by ChIP in the *htb1‐K123R* mutant (CY1272) and the corresponding WT strain (CY1256) after CR treatment (30 μg/mL). Results are expressed as the ratio between treated and untreated samples (CR+/CR−). (B) Ubi4‐HA enrichment at the *KDX1*‐BOX1 region was measured by ChIP in WT and *ubp8*Δ strains expressing Ubi4‐HA after 120 min and 240 min of CR treatment (30 μg/mL). Results are normalized to the WT (CR+/CR−), which was set to 1. Data correspond to the mean and standard deviation of at least three independent experiments.

How does Sus1 contribute to PIC formation and, consequently, transcriptional initiation? We have previously demonstrated that nucleosome displacement is essential for initiating gene transcription under conditions of cell wall stress [11, 14]. In this context, H2B monoubiquitination, catalyzed by Rad6/Bre1 and subsequently removed by Ubp8, appears to be essential for chromatin remodeling and efficient gene activation [15]. Since Sus1 facilitates the recruitment of Ubp8, as suggested by the requirement for both Sus1 and Ubp8 for their simultaneous recruitment to CWI‐dependent genes (Figure 3), we aimed to investigate the effect of deubiquitination on histone displacement at the *KDX1* promoter. We analyzed H3 and H2B histone displacement by ChIP in the htb1‐K123R mutant strain, which expresses a mutated H2B that cannot be ubiquitinated by Rad6, compared with the wild‐type strain. As shown in Figure 6A, the kinetics of H3 and H2B displacement in response to CR were similar for both strains, indicating that chromatin remodeling at the promoter region was not reliant on ubiquitination events. Consistent with this, the levels of ubiquitination at the *KDX1* promoter under stress conditions were not altered in an *ubp8*Δ strain compared to the WT strain (Figure 6B). Thus, Sus1 does not regulate PIC formation at the promoter of CWI‐dependent genes through histone H2B deubiquitination catalyzed by Ubp8.

**FIGURE 7:**
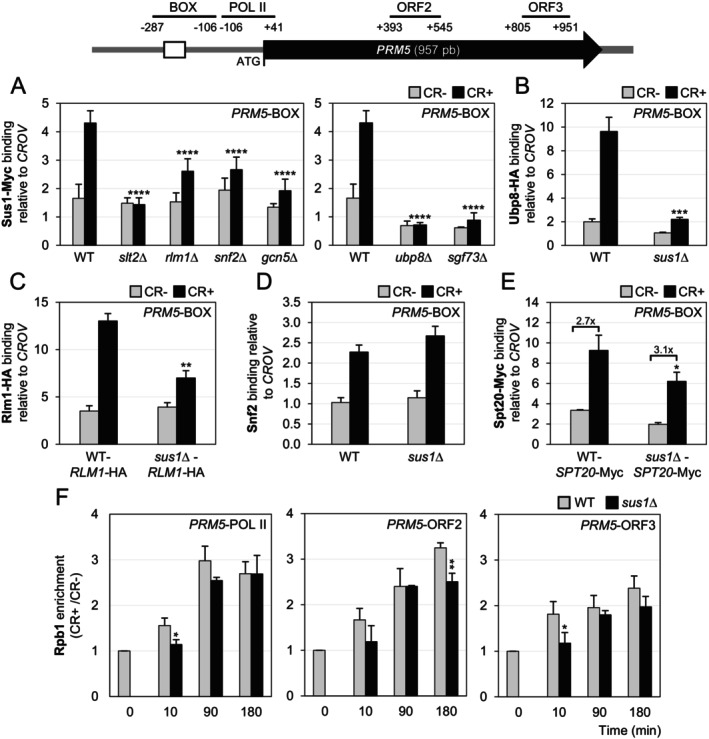
Sus1 recruitment to the *PRM5* gene and the effect of *SUS1* deletion on PIC assembly under cell wall stress. A schematic representation of the *PRM5* gene is depicted in the upper panel, with the Rlm1‐binding site marked by a white box. Regions amplified by primers for ChIP experiments are represented as horizontal lines, indicating their positions relative to the ATG start codon. (A) Sus1 binding to the *PRM5*‐BOX region was measured by ChIP in a WT strain and the indicated mutants expressing Sus1‐Myc after 3 h of CR addition (30 μg/mL). (B) ChIP assessed Ubp8 recruitment at the *PRM5*‐BOX region in WT and *sus1*Δ strains expressing epitope‐tagged Ubp8‐HA after CR treatment (3 h, 30 μg/mL). (C–E) Recruitment of Rlm1‐HA (C), Snf2 (D), and Spt20‐Myc (E) to the *PRM5* promoter (BOX region) was determined by ChIP using the corresponding WT and *sus1*Δ strains after CR treatment. (F) Pol II binding along the *PRM5* coding region was determined by ChIP with an anti‐Rpb1 antibody in the WT and *sus1*Δ strains after CR addition. Values represent the binding ratio between cells treated with CR and untreated cells. Data represent the mean and standard deviation of at least three independent experiments. Statistical significance was assessed using either ANOVA or Student's *t*‐test to compare mutants and the WT strain. Only significant differences are indicated: *p < 0.05, **p < 0.01, ***p < 0.001, and ****p < 0.0001.

At this point, we wondered if Sus1 was recruited to genes like *PRM5*, whose induction upon cell wall stress is Sus1‐independent. ChIP assays performed on a WT‐*SUS1*‐Myc strain revealed that Sus1 was also recruited to the *PRM5* promoter at levels similar to *KDX1* (Figure 7A). Moreover, this binding depended entirely on the presence of the MAPK Slt2 and Rlm1, although to a lesser extent, along with the coactivators SWI/SNF and SAGA (Figure 7A). Additionally, Sus1 binding required Ubp8 and Sgf73 (Figure 7A), and Ubp8 binds to the *PRM5* promoter in a Sus1‐dependent manner (Figure 7B), indicating that both subunits are co‐recruited as part of the SAGA complex to the target gene.

Furthermore, ChIP assays showed a decrease in Rlm1 recruitment in the *sus1*Δ strain compared to the wild type (Figure 7C). However, the recruitment of Snf2 and Spt20 under stress was not dependent on Sus1 at the *PRM5* promoter, with Spt20's recruitment differing from Sus1‐dependent genes (Figure 7D,E). Therefore, PIC formation was mostly unaffected in the absence of Sus1 at Sus1‐independent genes. Additionally, the recruitment of Pol II near the ATG at the *PRM5* promoter and its progression through the ORF were much less affected by the absence of Sus1 (Figure 7F) than in the cases of *KDX1* and *YPL088W*.

### 3.5 Sus1 and Gcn5 Collaborate in the Chromatin Remodeling Required to Promote SAGA‐Dependent Gene Expression in Response to Cell Wall Stress

**FIGURE 8:**
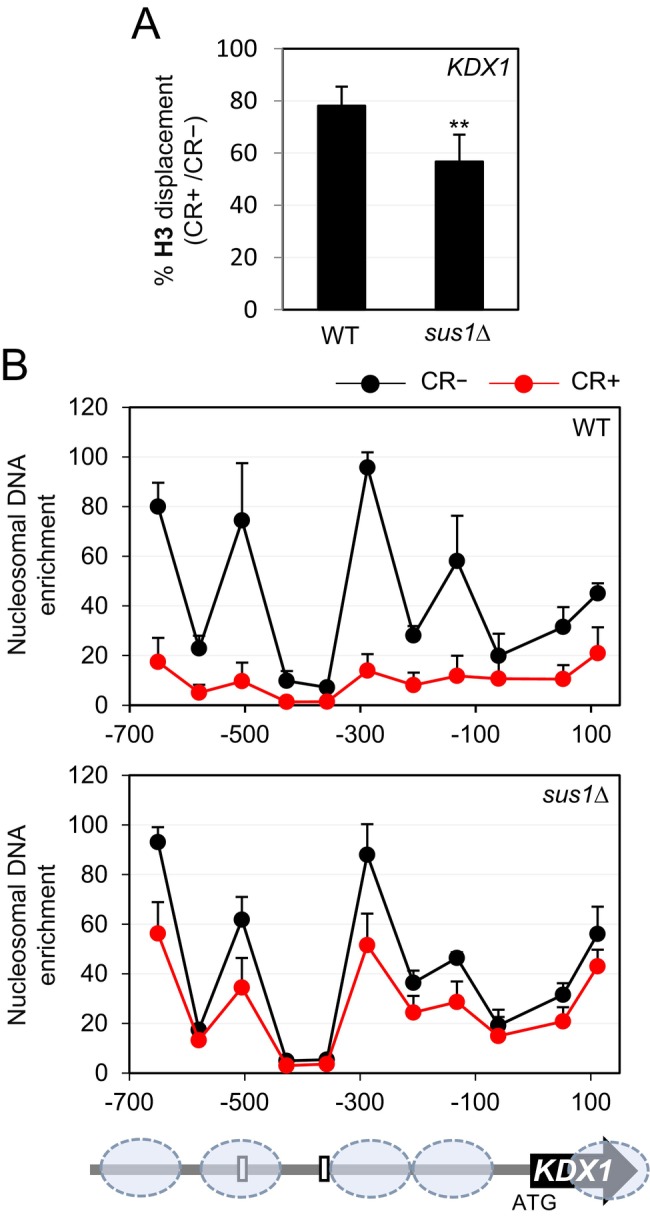
Histone H3 eviction and nucleosome displacement at CWI‐dependent genes in response to cell wall stress are defective in the absence of Sus1. (A) Histone H3 binding was analyzed by ChIP in WT and *sus1*Δ strains subjected to cell wall stress (CR 30 μg/mL, 3 h), at the promoter region of *KDX1* (BOX1). Results are shown as the percentage of H3 displacement (CR+/CR‐), with the basal histone enrichment in the WT strain set as 100% of H3 content. Data correspond to the mean and standard deviation from at least three independent experiments. Statistical analysis was conducted using a Student's *t*‐test, comparing the ratio of the *sus1*Δ mutant to the WT strain. Only statistically significant differences are indicated: **p < 0.01. (B) Nucleosome‐scanning analysis of the *KDX1* locus (from −699 to +161) in WT and *sus1*Δ strains under basal and cell wall stress conditions. Mononucleosomes were isolated from cultures grown with and without CR (3 h, 30 μg/mL). Purified DNA samples were analyzed by qPCR as described in Section 2. Nucleosomes are depicted as ovals at their corresponding positions in the *KDX1* locus, with predicted Rlm1‐binding sites shown as white boxes.

The remodeling of chromatin into a more open and relaxed conformation is crucial for the transcriptional machinery to access DNA and initiate transcription [10]. To determine whether the absence of Sus1 affected chromatin remodeling, chromatin from WT and *sus1*Δ strains, both exposed and not exposed to CR, was immunoprecipitated with an anti‐H3 antibody, and histone H3 enrichment at the *KDX1* promoter was assessed. Since the *sus1*Δ mutant showed lower levels of histone H3 under basal growth conditions, we expressed the results as a percentage of histone H3 eviction in each strain after CR exposure, allowing comparison between them. As shown in Figure 8A, CR treatment caused reduced H3 eviction at *KDX1* in the *sus1*Δ strain compared to the WT.

In light of these results, which suggested that Sus1 plays a role in chromatin remodeling during CWI pathway activation, we investigated its involvement in nucleosome displacement. We examined nucleosome positioning in the *KDX1* gene in wild‐type and *sus1*Δ strains before and after cell wall stress using micrococcal nuclease digestion of chromatin, followed by qPCR. As shown in Figure 8B and previously described [11], under basal conditions, the wild‐type strain exhibited four well‐positioned nucleosomes in the analyzed region of *KDX1*, with one occluding the Rlm1 binding box 2, one of the two Rlm1 binding sites at the *KDX1* promoter. Under stress conditions, this nucleosome pattern became completely disorganized. This remodeling was not observed in the *sus1*Δ strain (Figure 8B), where nucleosomes were slightly less positioned in response to stress, suggesting that Sus1 collaborates in nucleosome eviction during cell wall stress to activate *KDX1* gene expression.

**FIGURE 9:**
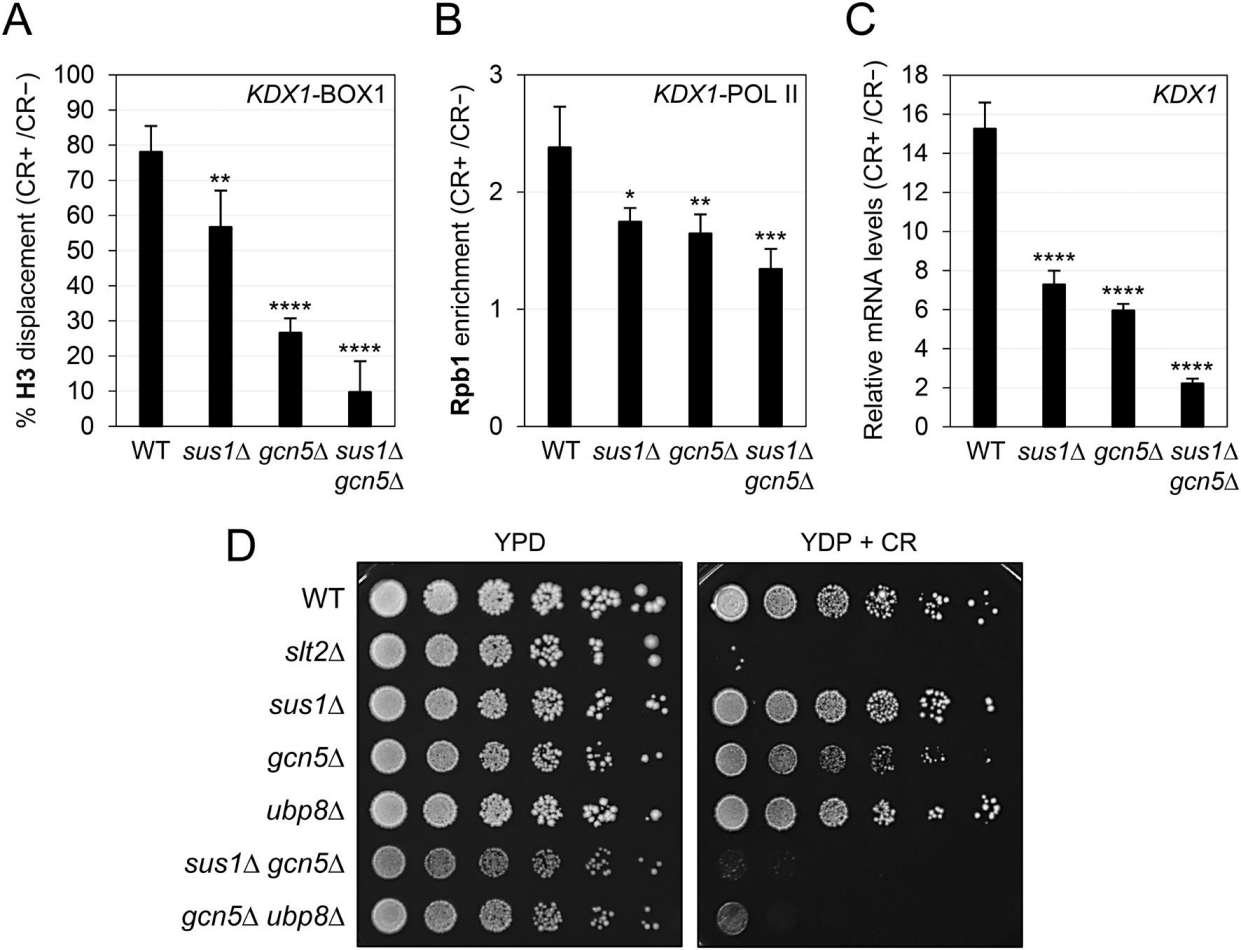
Sus1 and Gcn5 cooperate to remodel chromatin and regulate gene expression in response to cell wall stress. (A) The percentage of H3 displacement was measured by ChIP in the indicated strains after 3 h of CR treatment (30 μg/mL) at the promoter region (BOX1) of *KDX1*. (B) The binding of the Rpb1 subunit of Pol II to the POL II region of *KDX1* was analyzed in the indicated strains under the same conditions as in (A). (C) mRNA levels of *KDX1* were analyzed by RT‐qPCR in the same strains and conditions as in (A). Values show the mean and standard deviation of the ratio between CR‐treated and untreated samples from at least three independent experiments. The statistical analysis was performed using ANOVA to compare mutants and the WT strain (*p < 0.05, **p < 0.01, ***p < 0.001, and ****p < 0.0001). (D) Deletion of both *SUS1* and *GCN5* causes high hypersensitivity to CR. The indicated strains were spotted on YPD plates with or without 50 μg/mL of CR, and the plates were incubated for 48 h at 30°C.

Since the deletion of *SUS1* affects SAGA HATm recruitment to CWI‐dependent genes (Figure 4), Gcn5 is required for proper nucleosome displacement at these genes [14], and Gcn5 is part of the SAGA complex, we aimed to determine whether the impact on chromatin remodeling was caused by Sus1 itself or associated with the absence of Gcn5 in the promoter region of the CWI‐dependent genes. To explore this, we constructed the double mutant *gcn5*Δ *sus1*Δ. Histone H3 displacement at the *KDX1* promoter was measured by ChIP in WT, *sus1*Δ, *gcn5*Δ, and the double mutant *sus1*Δ *gcn5*Δ strains. As shown in Figure 9A, H3 displacement at *KDX1* promoters was more severely affected in the absence of Gcn5 than in the absence of Sus1; however, deleting both subunits together worsened this effect. This additive effect was also observed in the binding of the Rpb1 Pol II subunit (Figure 9B) and *KDX1* mRNA levels (Figure 9C). Consistent with a cooperative effect between Gcn5 and Sus1, even though the *sus1*Δ strain was not hypersensitive to CR (Figure 9D), the combined deletion of *SUS1* and *GCN*5 considerably aggravated the phenotype of the *gcn*5Δ strain, exhibiting a hypersensitive phenotype similar to that of the *slt2*Δ strain. Additionally, the phenotype of the double *gcn5*Δ *ubp8*Δ mutant strain resembled that of the double *sus1*Δ *gcn5*Δ mutant strain (Figure 9D), further supporting a prominent role of Sus1 in transcriptional activation rather than mRNA transport as part of the TREX‐2 complex. In summary, these findings demonstrate not only a direct role for Sus1 in chromatin remodeling of SAGA‐dependent genes in response to cell wall stress via the CWI pathway but also a cooperative interaction with Gcn5 in this process.

## 4 Discussion

Regulating gene expression to maintain cell viability during stress requires precise control over all aspects of mRNA fate, including transcription, mRNA processing, export to the cytoplasm, translation, and degradation, to ensure that cells respond appropriately and adapt to stress [57, 58, 59]. The transcription coactivator Spt‐Ada‐Gcn5‐acetyltransferase (SAGA) complex participates in various stages of this process [60, 61, 62, 63]. Previously, we demonstrated that the SAGA complex, through its histone acetylase activity mediated by Gcn5, works with the SWI/SNF complex to achieve the chromatin remodeling required for full gene expression in response to cell wall stress [4, 11, 14]. In addition to Gcn5, SAGA includes a deubiquitinase (Ubp8), which is part of the DUBm composed of Ubp8, Sus1, Sgf11, and Sgf73 [56, 64]. Here, we have delved into the characterization of the DUBm, establishing an additional role for Sus1, which is not only part of the DUBm but also of the nuclear pore‐associated TREX‐2 complex [24], in transcriptional activation through the CWI pathway. The requirement for Sus1 under conditions that perturb the cell wall through different mechanisms suggests that Sus1's effect on gene transcription via the CWI pathway is independent of the specific cell wall component affected, relying instead on the activation of the MAPK Slt2 in response to cell wall stress.

Our findings suggest that nearly all subunits of both complexes, including Sus1, are crucial for the induction of CWI‐dependent genes. Gene expression impairment was significantly more pronounced in TREX‐2 mutants, especially *sac3*Δ and *thp1*Δ strains, consistent with the role of these proteins within the complex. While Sac3 is essential for maintaining the integrity of the complex by acting as a scaffold that anchors the other subunits, Thp1 is involved in interactions with mRNAs [27, 65]. Although the TREX‐2 complex primarily participates in nucleocytoplasmic mRNA transport, several studies have shown that its subunits also regulate transcription, particularly during initiation and elongation, facilitating Sus1 binding to chromatin [32]. It is well established that the TREX‐2 complex is necessary for recruiting various modules of the SAGA complex to gene promoters during transcriptional activation and for the correct functioning of the DUBm itself [66]. Therefore, the effects observed in TREX‐2 complex mutants after exposure to compounds that induce cell wall damage may result from the combined impacts of gene mutations involved in both transcription and mRNA export. Deletion of *SAC3* led to decreased Rlm1 protein levels under cell wall stress, while *RLM1* transcript levels remained unchanged, suggesting that the TREX‐2 complex's influence on CWI gene expression might partly stem from defects in the nuclear export of *RLM1* transcripts. Since the positive feedback loop driven by the transcription factor Rlm1 on itself and on Slt2 [8] is essential for the CWI response and is disrupted in the absence of *SAC3*, we cannot exclude additional roles for TREX‐2 in transcriptional activation.

As Sus1 is a component of both the SAGA and TREX‐2 complexes, the impact of deleting *SUS1* on CWI‐dependent gene expression might stem from defects in transcriptional activation via SAGA and/or in the export of activated mRNAs through TREX‐2. Consistent with Sus1's dual role, our results show that Sus1 binding to CWI gene promoters depends on both Ubp8 and Sac3. However, while Rlm1 levels under stress conditions are reduced in a *sac3*Δ strain, they remain unchanged in a *sus1*Δ mutant. Additionally, Ubp8 associates with CWI‐dependent genes under stress in a Sus1‐dependent manner, whereas no association with Sac3 is observed under the same conditions, suggesting that Sus1 is recruited as part of the SAGA complex. While we cannot rule out that the transcriptional defect in response to stress in a *sus1*Δ strain is partly due to TREX‐2, these findings, along with the observation that there are only minor differences in the CR sensitivity phenotype between *sus1*Δ *gcn5*Δ and *ubp8*Δ *gcn5*Δ double mutants, suggest that under cell wall stress, Sus1 plays a more prominent role in transcriptional activation linked to SAGA rather than in mRNA export as part of the TREX‐2 complex.

Upon cell wall stress, SAGA acetylates histone H3 and collaborates with SWI/SNF to locally alter nucleosome positioning at the promoters of CWI‐responsive genes, thereby aiding PIC assembly [4, 11, 14]. We show here that cell wall stress directs Sus1, as part of the SAGA complex, to the promoters of these genes, requiring the MAPK Slt2, Rlm1, and coactivators SAGA and SWI/SNF. Although it is not essential for SWI/SNF recruitment, the binding of Rlm1, Spt20, and Pol II to CWI‐dependent gene promoters under stress is reduced in the absence of Sus1. The requirement of Sus1 for the association of the Spt20 subunit of SAGA complex with the promoters of CWI genes during stress contrasts with previous studies, which showed that SAGA's association with DNA during transcription activation is unaffected by the absence of DUBm components like Sus1 or Ubp8 [18, 67].

Based on our previous results, SAGA recruitment to CWI‐responsive genes relies on the SWI/SNF complex, whereas Gcn5 is largely unnecessary for Snf2 recruitment [14]. This suggests that SWI/SNF may be recruited by Rlm1 before SAGA, allowing its subsequent entry. Consistent with this, our findings show that Sus1 associates with promoters as part of the SAGA complex and that its recruitment depends on SWI/SNF, while Sus1 is not required for Snf2 binding to CWI‐dependent genes. These observations support a model in which Sus1 is recruited as part of SAGA downstream of SWI/SNF activity. However, as previously discussed [14], we cannot rule out the simultaneous entry of both complexes. In this scenario, the SWI/SNF complex might play a more significant role in chromatin remodeling.

Our findings indicate that Sus1 plays a crucial role in the association of Pol II with Sus1‐dependent genes and in the progression along the coding regions of these genes. Consistent with this, a high percentage of the CWI transcriptional response (70%) triggered by cell wall stress depends on Sus1. Unlike the minimal overlap between Sus1‐dependent genes and gene regulation by other SAGA subunits (Gcn5, Spt3, and Spt20) under normal growth conditions [24, 68], we observed a significant correlation between the regulation of gene expression by Sus1 and Gcn5 [14] under cell wall stress. Both subunits co‐regulate 56% of the genes induced in a WT strain under these conditions, suggesting an essential overlap between different modules of the SAGA complex in regulating gene expression during stress. Few studies have explored the role of Sus1 under stress conditions. Sus1 preferentially relocates to promoters of SAGA‐dominated genes, especially those involved in the environmental stress response (ESR), during heat shock [36]. Although cell wall stress does not trigger the typical ESR, our data suggest that other stresses, such as cell wall stress, activate the recruitment of Sus1 and other SAGA subunits, including Gcn5 [14], to CWI‐dependent genes, thereby influencing transcriptional activation.

How does Sus1 contribute to PIC formation and transcription initiation during cell wall stress? Since Sus1 facilitates the recruitment of Ubp8 to CWI‐dependent genes under stress conditions, this effect might be related to H2B deubiquitination by Ubp8. However, the kinetics of H3 and H2B displacement in response to cell wall stress in an htb1‐K123R mutant strain—which produces an H2B that Rad6 cannot ubiquitinate—were similar to those in a wild‐type strain. Additionally, no significant differences in ubiquitination levels of CWI‐dependent gene promoters were observed between *ubp8*Δ and wild‐type strains exposed to CR, indicating that Sus1's effect on PIC formation under these conditions is not mediated by H2B deubiquitination. Previous studies have shown that Sus1 also interacts with the promoters of *GAL1* and other SAGA‐dependent genes such as *ADH1*, *ARG1*, and *PHO84* during transcriptional activation, aiding in PIC formation [18, 24, 31, 32, 56, 69, 70]. For *GAL1*, *ADH1*, and *PHO84*, this role is also independent of histone H2B deubiquitination, as losing Ubp8 does not affect the recruitment of TBP and Rpb1 to their core promoters after transcriptional induction [18, 31, 69]. In the case of *GAL1*, it has been proposed that Sus1 influences PIC formation by promoting Sgf73 recruitment to the *GAL1* UAS, which is essential for the overall structural integrity of the SAGA complex. Conversely, the association of activator Gal4 with the *GAL1* UAS remains unchanged in the *sus1*Δ strain. This differs somewhat from what we observe under cell wall stress, where Rlm1's recruitment to CWI‐dependent genes decreases in the absence of Sus1. Moreover, Gcn5 is not necessary for PIC assembly at *GAL1* [18, 71], but at CWI‐dependent genes, this process relies on the HAT activity of SAGA [14], indicating subtle differences in gene regulation via SAGA under various activation conditions.

The SAGA complex is responsible for increasing gene expression under cell wall stress through H3 histone acetylation [14]. We have previously suggested that SAGA does not mediate TBP delivery to *KDX1*, or at least not as exclusively as it does for other stress‐responsive genes, since deleting *SPT3* or *SPT8*, which mediates this process, does not cause cell wall stress hypersensitivity [14]. Here, we demonstrate that Sus1 is involved in chromatin remodeling during CWI pathway‐mediated transcriptional activation. Although deleting *SUS1* does not affect the binding of the SWI/SNF complex, H3 eviction and nucleosome displacement at CWI‐dependent gene promoters under stress are severely impaired in a *sus1*Δ strain. Another SAGA DUBm subunit, Sgf73, which connects the DUBm to the rest of the SAGA complex and is also necessary for Sus1's association with the SAGA and TREX‐2 complexes—and for NPC‐TREX‐2 stabilization—has also been linked to chromatin disorganization and the release of histone H3 from the promoters of various SAGA‐dependent genes during transcriptional activation [69, 72].

Since histone eviction at CWI‐dependent gene promoters is unaffected by H2B deubiquitination and Gcn5 is essential for effective nucleosome removal at these genes [14], the role of Sus1 in chromatin remodeling could be mediated either directly by Sus1 itself or through its association with the HAT module via Gcn5's catalytic activity. A compromised overall structural integrity of SAGA in the absence of Sus1 would reduce SAGA's HAT activity. However, analysis of a double *sus1*Δ *gcn5*Δ mutant strain showed an additive effect of Gcn5 and Sus1 on chromatin remodeling, indicating that Sus1´s involvement in this process is independent of Gcn5‐mediated histone acetylation. Therefore, Sus1 may play a direct function working with SAGA's HATm to displace nucleosomes at CWI‐dependent genes, facilitating Pol II entry and progression. How Sus1 promotes nucleosome remodeling remains unclear; it might stabilize the interaction between SAGA and chromatin or interact with other remodelers or coactivators. Supporting this, the structure of Sus1 complexed with TREX‐2 or SAGA shows that Sus1 has an articulated helical hairpin fold. Despite its small size, its extended fold creates a large surface area that enables multiple associations with other components of the gene expression machinery [30, 73].

Interestingly, although Sus1 is not necessary for the transcriptional activation of specific CWI‐responsive genes, like *PRM5*, it is still recruited to their promoters in a manner dependent on Slt2, Rlm1, SWI/SNF, and SAGA. While the absence of Sus1 moderately reduces Rlm1 recruitment, it does not affect the assembly of other PIC components, such as SAGA and Pol II, nor does it impact the expression of these genes. This probably reflects differences in promoter architecture. The *PRM5* promoter has a larger NFR that contains its unique Rlm1‐binding site [74, 75, 76]. In this context, nucleosome eviction may be less important than at promoters, where Rlm1 binding sites are occluded by nucleosomes, allowing Pol II to access and move along coding regions without additional regulators. During cell wall stress, other SAGA components like Gcn5 are also recruited to genes where they do not influence gene expression [14], although their precise role remains unclear.

In addition to promoting transcription initiation, Sus1 is recruited to the coding regions of CWI‐dependent genes, suggesting a possible role in supporting transcriptional elongation. Although this aspect was not extensively explored in our study, it aligns with previous reports describing how Sus1 facilitates transcriptional elongation through its interaction with the elongating form of RNA Pol II and other factors involved in mRNA export [32].

In summary, our study uncovers novel regulatory mechanisms through which the MAPK Slt2 controls gene expression in *Saccharomyces cerevisiae* and highlights the key role of Sus1 in chromatin remodeling and transcriptional control of the yeast CWI stress response. Since yeast cell wall stress responses may reduce the effectiveness of antifungal treatments targeting the cell wall [77, 78], our findings could improve understanding of transcriptional regulation relevant to antifungal development. Moreover, MAPK signaling and chromatin‐associated transcriptional regulation mechanisms are highly conserved in humans and are functionally linked to various human diseases [79], underscoring the relevance of our results for human health.

## Author Contributions

**M.P.‐V.:** visualization, methodology, investigation, formal analysis. **R.G.:** visualization, methodology, investigation, formal analysis. **J.M.R.**‐**P.:** investigation, formal analysis, supervision. **A.B.S.:** methodology, investigation, formal analysis, writing – review and editing, writing – original draft, supervision, conceptualization. **J.A.:** writing – review and editing, writing – original draft, formal analysis, supervision, funding acquisition, conceptualization. All authors read and approved the final manuscript.

## Funding

This work was supported by grants PID2019‐105223GB‐I00 and PID2022‐136888NB‐I00, funded by MCIN/AEI/10.13039/501100011033 (Ministerio de Ciencia e Innovación/Agencia Estatal de Investigación, Spain) to J.A.

## Conflicts of Interest

The authors declare no conflicts of interest.

## Supporting information


**Figure S1:** Slt2 phosphorylation levels in the absence of Sus1 and subunits of the SAGA DUBm and TREX‐2 complexes under cell wall stress. (A, B) Phospho‐Slt2 (P‐Slt2) and total Slt2 protein levels were analyzed by Western blot using anti‐phospho‐p44/42 MAPK and anti‐Slt2 antibodies, respectively, in WT and mutant strains lacking Sus1 (A) and subunits of the DUBm SAGA (B, left) and TREX‐2 complexes (B, right) after 3 h of CR treatment (30 μg/mL). G6PDH was used as a loading control. Graphs depict quantification of Slt2 activation by densitometric analysis of P‐Slt2 relative to total Slt2 protein in each condition, normalized to G6PDH bands, with WT strain levels in the absence of stress set as the reference (fold change = 1.0). Data are presented as the mean and standard deviation of three independent experiments. Statistical significance was assessed using Student's *t*‐test or ANOVA, comparing mutant to the WT strain. Only significant differences are shown: *p < 0.05, **p < 0.01, ***p < 0.001, and ****p < 0.0001.
**Figure S2:** Expression of CWI‐responsive genes is reduced in the *sus1*Δ mutant under other cell wall stress conditions. *KDX1* and *PIR3* mRNA levels were analyzed by RT‐qPCR in WT and *sus1*Δ strains after treatment with zymolyase (0.8 U/mL, 2 h) (A) or caspofungin (15 ng/mL, 2 h) (B). Values represent the ratio between CR‐treated and untreated cells. Data are presented as the mean and standard deviation from at least three independent experiments. Statistical significance was assessed using Student's *t*‐test, comparing the mutant to the WT strain. Only significant differences are shown: *p < 0.05, **p < 0.01, ***p < 0.001, and ****p < 0.0001.
**Figure S3:** Rlm1 levels under cell wall stress in the absence of Sus1, Ubp8 (SAGA DUBm), and Sac3 (TREX‐2) subunits. (A) The levels of Rlm1 and phospho‐Slt2 (P‐Slt2) were analyzed by Western blot using anti‐HA and anti‐phospho‐p44/42 MAPK antibodies in WT and *sus1*Δ strains expressing the epitope‐tagged Rlm1‐HA protein after 3 h of CR treatment (30 μg/mL). G6PDH served as a loading control. Graphs depict the fold change in Rlm1 protein levels, determined by densitometric quantification of the Rlm1‐HA bands, normalized against G6PDH. The value for the WT strain grown without stress was set as a reference (fold change = 1.0). (B) The levels of Rlm1 and P‐Slt2 were analyzed by Western blot after 3 h of CR treatment (30 μg/mL) using anti‐Myc and anti‐phospho‐p44/42 MAPK antibodies in WT and *sus1*Δ strains expressing the epitope‐tagged Rlm1‐Myc protein, with or without an additional copy of Rlm1‐Myc from a multicopy plasmid (YEp181‐*RLM1*‐6xMyc). Rlm1 quantification was performed as described in (A), and the corresponding graphs are presented. (C) Relative mRNA levels of the *RLM1* gene (CR+/CR) were analyzed by RT‐qPCR in WT and *sac3*Δ strains after 3 h of CR treatment. Data correspond to the mean and standard deviation of at least three independent experiments. Statistical significance was assessed using Student's *t*‐test, comparing mutant to the WT strain. Only significant differences are shown: *p < 0.05, **p < 0.01, ***p < 0.001, and ****p < 0.0001.
**Figure S4:** Sac3, a component of the TREX‐2 complex, is not recruited to the promoter of Sus1‐dependent genes. Sac3 recruitment at the *KDX1*‐BOX1 and *YPL088W*‐BOX regions was determined by ChIP in a WT strain expressing the epitope‐tagged Sac3‐Myc protein after 3 h of CR addition (30 μg/mL). Data correspond to the mean and standard deviation of at least three independent experiments. Statistical significance was assessed using Student's *t*‐test, comparing mutant to the WT strain. Only significant differences are shown: *p < 0.05, **p < 0.01, ***p < 0.001, and ****p < 0.0001.

**Table S1:** Yeast strains used in this study.

**Table S2:** Primers used in this work.

**Table S3:** List of genes induced and repressed under CR treatment (30 μg/mL, 3 h) in a WT strain and its dependence on Sus1 and Gcn5.

## Data Availability

The microarray data described here have been deposited in the NCBI Gene Expression and Hybridization Array Data Repository under accession GSE301493. Other data supporting the findings of this study are included in this published article and its Supporting Information. Materials are available upon request from the corresponding authors.
