# Supplementary material for: Sus1 Modulates Chromatin Remodeling and Gene Expression via the Cell Wall Integrity Pathway in Saccharomyces cerevisiae
Source: FASEB J. 2026 Apr 28;40:e71848. doi: 10.1096/fj.202504656RR (PMC13123632; doi:10.1096/fj.202504656RR)
Supplement: Supplementary file 1 — Figure S1: Slt2 phosphorylation levels in the absence of Sus1 and subunits of the SAGA DUBm and TREX‐2 complexes under cell wall stress. (A, B) Phospho‐Slt2 (P‐Slt2) and total Slt2 protein levels were analyzed by Western blot using anti‐phospho‐p44/42 MAPK and anti‐Slt2 antibodies, respectively, in WT and mutant strains lacking Sus1 (A) and subunits of the DUBm SAGA (B, left) and TREX‐2 complexes (B, right) after 3 h of CR treatment (30 μg/mL). G6PDH was used as a loading control. Graphs depict quantification of Slt2 activation by densitometric analysis of P‐Slt2 relative to total Slt2 protein in each condition, normalized to G6PDH bands, with WT strain levels in the absence of stress set as the reference (fold change = 1.0). Data are presented as the mean and standard deviation of three independent experiments. Statistical significance was assessed using Student's t‐test or ANOVA, comparing mutant to the WT strain. Only significant differences are shown: *p < 0.05, **p < 0.01, ***p < 0.001, and ****p < 0.0001. Figure S2: Expression of CWI‐responsive genes is reduced in the sus1Δ mutant under other cell wall stress conditions. KDX1 and PIR3 mRNA levels were analyzed by RT‐qPCR in WT and sus1Δ strains after treatment with zymolyase (0.8 U/mL, 2 h) (A) or caspofungin (15 ng/mL, 2 h) (B). Values represent the ratio between CR‐treated and untreated cells. Data are presented as the mean and standard deviation from at least three independent experiments. Statistical significance was assessed using Student's t‐test, comparing the mutant to the WT strain. Only significant differences are shown: *p < 0.05, **p < 0.01, ***p < 0.001, and ****p < 0.0001. Figure S3: Rlm1 levels under cell wall stress in the absence of Sus1, Ubp8 (SAGA DUBm), and Sac3 (TREX‐2) subunits. (A) The levels of Rlm1 and phospho‐Slt2 (P‐Slt2) were analyzed by Western blot using anti‐HA and anti‐phospho‐p44/42 MAPK antibodies in WT and sus1Δ strains expressing the epitope‐tagged Rlm1‐HA protein after 3 h [file FSB2-40-e71848-s002.pptx]

## Slide 1
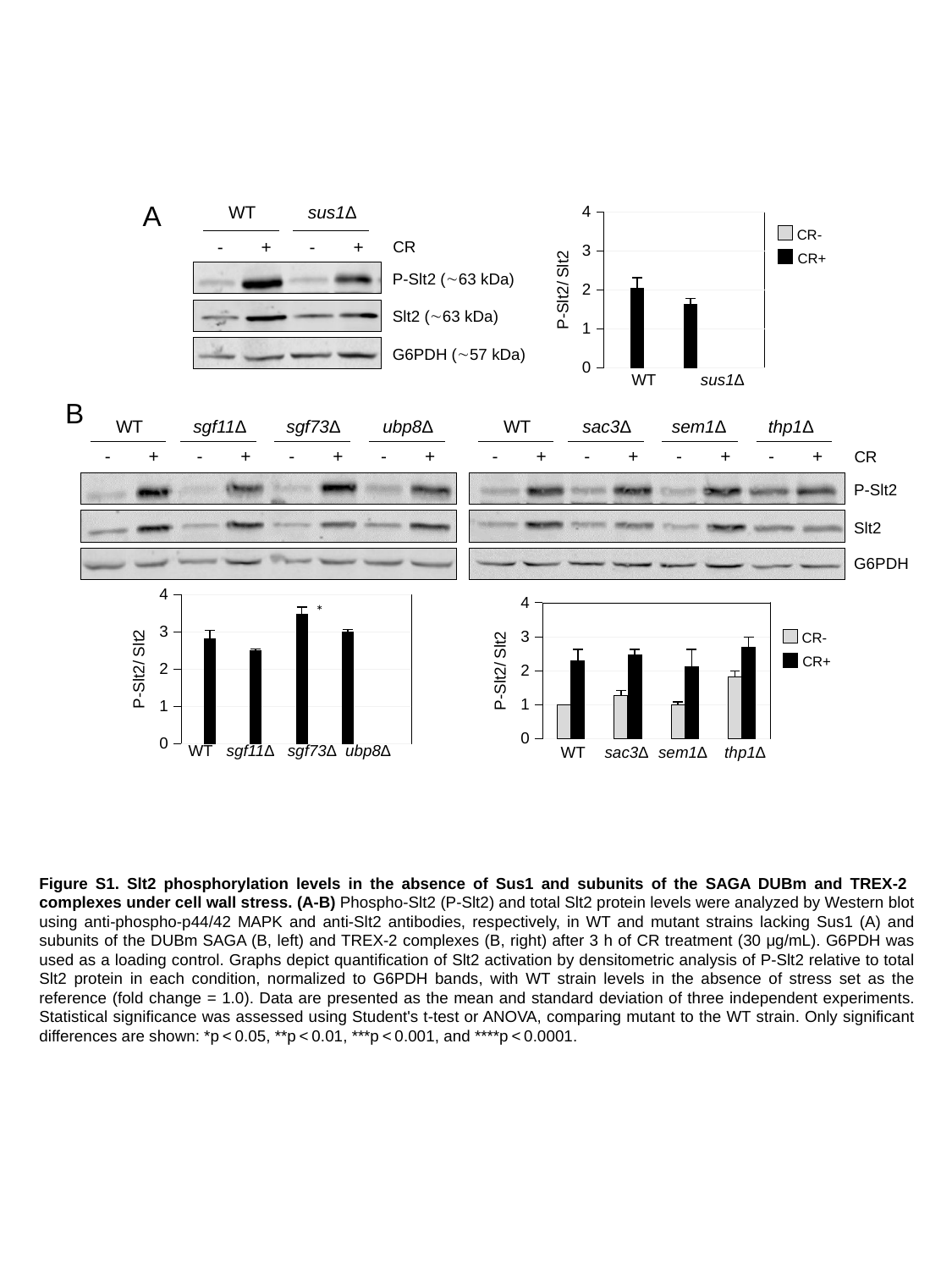

A
### Chart
| Category | RC+ | RC- |
|---|---|---|
| WT | 1.0 | 2.0500307890146465 |
| Sus1 | 0.5781244850436287 | 1.6209637816609677 |CR-
CR+
sus1∆
WT
WT
sus1Δ
-
+
-
+
CR
P-Slt2 (63 kDa)
Slt2 (63 kDa)
G6PDH (57 kDa)
B
WT
sgf11Δ
sgf73Δ
ubp8Δ
WT
sac3Δ
sem1Δ
thp1Δ
-
+
-
+
-
+
-
+
-
+
-
+
-
+
-
+
CR
P-Slt2
Slt2
G6PDH
### Chart
| Category | RC+ | RC- |
|---|---|---|
| WT | 1.0 | 2.815560382357834 |
| Sgf11∆ | 1.4880318885520654 | 2.4968883063547747 |
| Sgf73∆ | 1.5524985921187495 | 3.4678567772820097 |
| Ubp8∆ | 1.4267650884115335 | 2.9831836852114852 |sgf11∆
sgf73∆
ubp8∆
WT
### Chart
| Category | RC+ | RC- |
|---|---|---|
| WT | 1.0 | 2.300772570954448 |
| sac3∆ | 1.272298235684105 | 2.465382410197557 |
| sem1∆ | 0.9932738222031278 | 2.119218574990352 |
| thp1∆ | 1.82716796523182 | 2.6925287265302464 |CR-
CR+
sac3∆
sem1∆
thp1∆
WT
*
Figure S1. Slt2 phosphorylation levels in the absence of Sus1 and subunits of the SAGA DUBm and TREX-2 complexes under cell wall stress. (A-B) Phospho-Slt2 (P-Slt2) and total Slt2 protein levels were analyzed by Western blot using anti-phospho-p44/42 MAPK and anti-Slt2 antibodies, respectively, in WT and mutant strains lacking Sus1 (A) and subunits of the DUBm SAGA (B, left) and TREX-2 complexes (B, right) after 3 h of CR treatment (30 μg/mL). G6PDH was used as a loading control. Graphs depict quantification of Slt2 activation by densitometric analysis of P-Slt2 relative to total Slt2 protein in each condition, normalized to G6PDH bands, with WT strain levels in the absence of stress set as the reference (fold change = 1.0). Data are presented as the mean and standard deviation of three independent experiments. Statistical significance was assessed using Student's t-test or ANOVA, comparing mutant to the WT strain. Only significant differences are shown: *p < 0.05, **p < 0.01, ***p < 0.001, and ****p < 0.0001.

## Slide 2
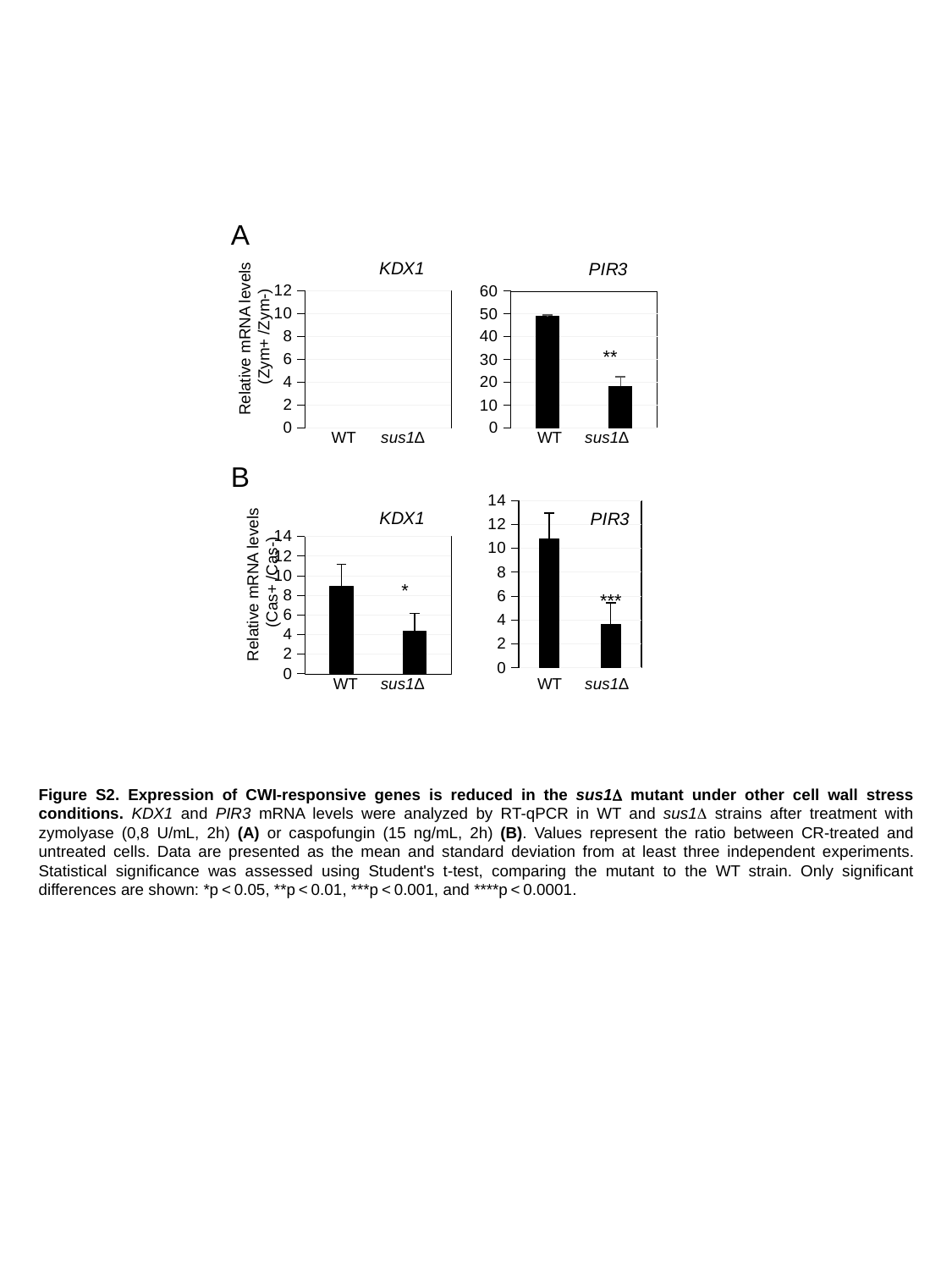

A
### Chart: KDX1
| Category | ZYM+/ZYM- |
|---|---|
| WT | 21.237182499262964 |
| Sus1∆ | 16.574755370480702 |Relative mRNA levels
 (Zym+ /Zym-)
WT
sus1∆
### Chart: PIR3
| Category | ZYM+/ZYM- |
|---|---|
| WT | 48.90484649123141 |
| Sus1∆ | 18.23488887307246 |**
WT
sus1∆
B
### Chart: KDX1
| Category | MLP1 |
|---|---|
| WT | 8.89514224361701 |
| sus1Δ | 4.3478187480762 |Relative mRNA levels
 (Cas+ /Cas-)
WT
sus1∆
### Chart: PIR3
| Category | PIR3 |
|---|---|
| WT | 10.76771848562344 |
| sus1Δ | 3.6221919811511443 |*
***
WT
sus1∆
Figure S2. Expression of CWI-responsive genes is reduced in the sus1Δ mutant under other cell wall stress conditions. KDX1 and PIR3 mRNA levels were analyzed by RT-qPCR in WT and sus1Δ strains after treatment with zymolyase (0,8 U/mL, 2h) (A) or caspofungin (15 ng/mL, 2h) (B). Values represent the ratio between CR-treated and untreated cells. Data are presented as the mean and standard deviation from at least three independent experiments. Statistical significance was assessed using Student's t-test, comparing the mutant to the WT strain. Only significant differences are shown: *p < 0.05, **p < 0.01, ***p < 0.001, and ****p < 0.0001.

## Slide 3
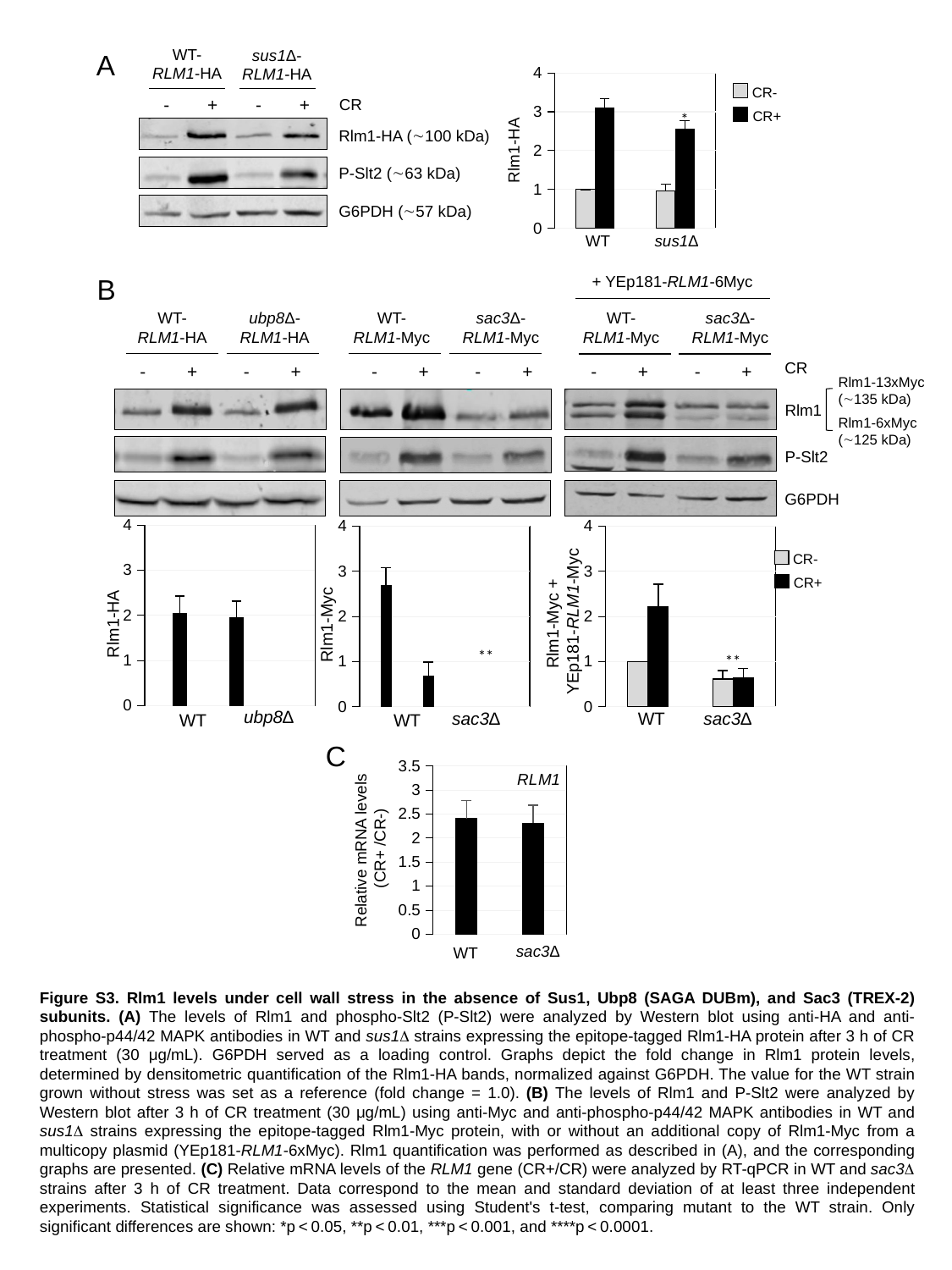

WT-
RLM1-HA
sus1Δ-
RLM1-HA
A
### Chart
| Category | RC+ | RC- |
|---|---|---|
| WT | 1.0 | 3.096303581523655 |
| Sus1 | 0.9634819762312822 | 2.5585388179561743 |CR-
CR+
sus1∆
WT
-
+
-
+
CR
*
Rlm1-HA (100 kDa)
P-Slt2 (63 kDa)
G6PDH (57 kDa)
B
+ YEp181-RLM1-6Myc
WT-
RLM1-HA
ubp8Δ-
RLM1-HA
WT-
RLM1-Myc
sac3Δ-
RLM1-Myc
WT-
RLM1-Myc
sac3Δ-
RLM1-Myc
CR
-
+
-
+
-
+
-
+
-
+
-
+
Rlm1-13xMyc (135 kDa)
Rlm1
Rlm1-6xMyc (125 kDa)
### Chart
| Category | RC- | RC+ |
|---|---|---|
| WT | 1.0 | 2.6808080629149242 |
| sac3∆ | 0.45153487293537525 | 0.6809103450543471 |Rlm1-Myc
**
sac3∆
WT
P-Slt2
G6PDH
### Chart
| Category | RC- | RC+ |
|---|---|---|
| WT | 1.0 | 2.038187522565924 |
| ubp8 | 1.0842339906461478 | 1.953782879717474 |Rlm1-HA
ubp8∆
WT
### Chart
| Category | RC- | RC+ |
|---|---|---|
| WT | 1.0 | 2.2183806438971034 |
| sac3∆ | 0.6142522514797183 | 0.6378022750601442 |CR-
CR+
Rlm1-Myc +
 YEp181-RLM1-Myc
**
sac3∆
WT
C
### Chart: RLM1
| Category | CR+/CR- |
|---|---|
| WT | 2.4073230459234805 |
| sac3∆ | 2.3124980383360483 |sac3∆
WT
Relative mRNA levels
 (CR+ /CR-)
Figure S3. Rlm1 levels under cell wall stress in the absence of Sus1, Ubp8 (SAGA DUBm), and Sac3 (TREX-2) subunits. (A) The levels of Rlm1 and phospho-Slt2 (P-Slt2) were analyzed by Western blot using anti-HA and anti-phospho-p44/42 MAPK antibodies in WT and sus1Δ strains expressing the epitope-tagged Rlm1-HA protein after 3 h of CR treatment (30 μg/mL). G6PDH served as a loading control. Graphs depict the fold change in Rlm1 protein levels, determined by densitometric quantification of the Rlm1-HA bands, normalized against G6PDH. The value for the WT strain grown without stress was set as a reference (fold change = 1.0). (B) The levels of Rlm1 and P-Slt2 were analyzed by Western blot after 3 h of CR treatment (30 μg/mL) using anti-Myc and anti-phospho-p44/42 MAPK antibodies in WT and sus1Δ strains expressing the epitope-tagged Rlm1-Myc protein, with or without an additional copy of Rlm1-Myc from a multicopy plasmid (YEp181-RLM1-6xMyc). Rlm1 quantification was performed as described in (A), and the corresponding graphs are presented. (C) Relative mRNA levels of the RLM1 gene (CR+/CR) were analyzed by RT-qPCR in WT and sac3Δ strains after 3 h of CR treatment. Data correspond to the mean and standard deviation of at least three independent experiments. Statistical significance was assessed using Student's t-test, comparing mutant to the WT strain. Only significant differences are shown: *p < 0.05, **p < 0.01, ***p < 0.001, and ****p < 0.0001.

## Slide 4
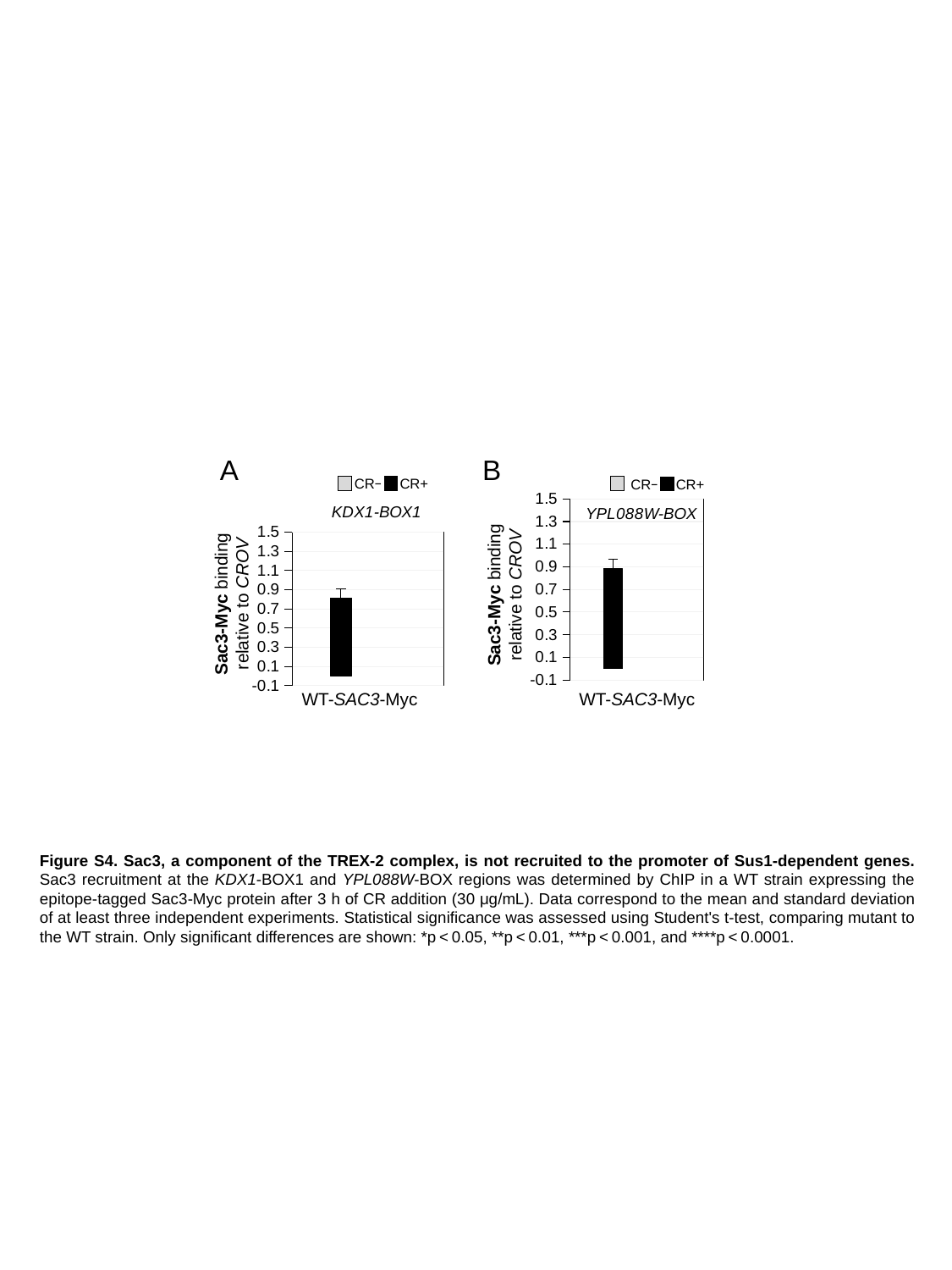

A
B
CR−
CR+
CR−
CR+
### Chart: YPL088W-BOX
| Category | RC- | RC+ |
|---|---|---|
| WT-Sac3-Myc | 0.7424233105663269 | 0.8824914229259161 |Sac3-Myc binding relative to CROV
WT-SAC3-Myc
### Chart: KDX1-BOX1
| Category | RC- | RC+ |
|---|---|---|
| WT-Sac3-Myc | 0.789209646193837 | 0.8118248114292159 |Sac3-Myc binding relative to CROV
WT-SAC3-Myc
Figure S4. Sac3, a component of the TREX-2 complex, is not recruited to the promoter of Sus1-dependent genes. Sac3 recruitment at the KDX1-BOX1 and YPL088W-BOX regions was determined by ChIP in a WT strain expressing the epitope-tagged Sac3-Myc protein after 3 h of CR addition (30 μg/mL). Data correspond to the mean and standard deviation of at least three independent experiments. Statistical significance was assessed using Student's t-test, comparing mutant to the WT strain. Only significant differences are shown: *p < 0.05, **p < 0.01, ***p < 0.001, and ****p < 0.0001.
