# Supplementary material for: Sus1 Modulates Chromatin Remodeling and Gene Expression via the Cell Wall Integrity Pathway in Saccharomyces cerevisiae
Source: FASEB J. 2026 Apr 28;40:e71848. doi: 10.1096/fj.202504656RR (PMC13123632; doi:10.1096/fj.202504656RR)
Supplement: Supplementary file 2 — Table S1: Yeast strains used in this study. [file FSB2-40-e71848-s001.docx]

| **Strain** | **Genotype** | **Source or reference** |
| --- | --- | --- |
| BY4741 | *MAT***a**; *his3*Δ*1; leu2*Δ*0; met15*Δ*0; ura3*Δ*0* | Euroscarf (Y00000) |
| *sac3*Δ | BY4741; *sac3::KanMX4* | Euroscarf (Y03517) |
| *sem1*Δ | BY4741; *sem1::KanMX4* | Euroscarf (Y05828) |
| *thp1*Δ | BY4741; *thp1::KanMX4* | Euroscarf (Y01764) |
| *sgf11*Δ | BY4741; *sgf11::KanMX4* | Euroscarf (Y02781) |
| *sgf73*Δ | BY4741; *sgf73::KanMX4* | Euroscarf (Y04433) |
| *ubp8*Δ | BY4741; *ubp8::KanMX4* | Euroscarf (Y00809) |
| *gcn5*Δ | BY4741; *gcn5::KanMX4* | Euroscarf (Y07285) |
| *slt2*Δ | BY4741; *slt2::KanMX4* | Euroscarf (Y00993) |
| *sus1*Δ | BY4741; *sus1::KanMX4* | This work |
| *sus1*Δ *gcn5*Δ | *MAT***a**; *his3*Δ*1; leu2*Δ*0; met15*Δ*0; ura3*Δ*0; lys2*Δ*0; sus1::KanMX4; gcn5::KanMX4* | This work |
| *gcn5*Δ *ubp8*Δ | BY4741*; GCN5::KanMX6; UBP8::KanMX6* | Jerry L. Workman (Lee *et al.*, 2005) |
| WT*-RLM1-*HA | BY4741; *RLM1-3xHA::LEU2* | Sanz *et al*., 2012 |
| *sus1*Δ-*RLM1-*HA | BY4741; *sus1::KanMX4; RLM1-3xHA::LEU2* | This work |
| *ubp8*Δ-*RLM1-*HA | BY4741; *ubp8::KanMX4; RLM1-3xHA::LEU2* | This work |
| WT-*RLM1*-Myc | BY4741; *RLM1-13xMyc::HIS3* | García *et al*., 2016 |
| *sac3*Δ*-RLM1-*Myc | BY4741; *sac3::KanMX4; RLM1-13xMyc::HIS3* | This work |
| WT-*SUS1*-Myc | BY4741; *SUS1-13xMyc::HIS3* | This work |
| *slt2*Δ*-SUS1-*Myc | BY4741; *slt2::KanMX4; SUS1-13xMyc::HIS3* | This work |
| *rlm1*Δ*-SUS1-*Myc | BY4741; *rlm1::KanMX4; SUS1-13xMyc::HIS3* | This work |
| *sac3*Δ*-SUS1-*Myc | BY4741; *sac3::KanMX4; SUS1-13xMyc::HIS3* | This work |
| *ubp8*Δ*-SUS1-*Myc | BY4741; *ubp8::KanMX4; SUS1-13xMyc::HIS3* | This work |
| *sgf73*Δ*-SUS1-*Myc | BY4741; *sgf73::KanMX4; SUS1-13xMyc::HIS3* | This work |
| *gcn5*Δ*-SUS1-*Myc | BY4741; *gcn5::KanMX4; SUS1-13xMyc::HIS3* | This work |
| *snf2*Δ*-SUS1-*Myc | BY4741; *snf2::KanMX4; SUS1-13xMyc::HIS3* | This work |
| WT-*UBP8-*HA | BY4741; *UBP8-3xHA::HIS3* | Susana Rodríguez-Navarro (Köhler *et al.*, 2006) |
| *sus1*Δ-*UBP8-*HA | BY4741; *sus1::KanMX4; UBP8-3xHA::HIS3* | This work |
| WT-*SPT20*-Myc | BY4741; *SPT20-13xMyc::KanMX4* | Sanz *et al*., 2016 |
| *sus1*Δ*-SPT20-*Myc | BY4741; *sus1::HIS3; SPT20-13xMyc::KanMX4* | This work |
| WT-*SAC3*-Myc | BY4741; *SAC3-13xMyc::HIS3* | This work |
| CY1256 | BY4741; *htb2*Δ*0* | Christopher J. Brandl (Turner *et al*., 2002) |
| CY1272 | BY4741; *HTB1K123R; htb2*Δ*0* | Christopher J. Brandl (Turner *et al.*, 2002) |
| RGY5566 | BY4741; *pTDH3-3xHA-Ub::URA3* | Richard Gardner |
| *ubp8*Δ (*pTDH3-3xHA-Ub*) | BY4741; *pTDH3-3xHA-Ub::URA3; ubp8::KanMX4* | This work |

**Table S1: Yeast strains used in this study.**

-R. García, A. B. Sanz, J. M. Rodríguez-Peña, C. Nombela, J. Arroyo (2016). J Cell Sci 129:1649-1660. https://doi.org/10.1242/jcs.180190.

-K. K. Lee, L. Florens, S. K. Swanson, M. P. Washburn, J. L. Workman (2005). Mol Cell Biol 25:1173-1182. https://doi.org/10.1128/MCB.25.3.1173-1182.2005.

-A. B. Sanz, R. García, J. M. Rodríguez-Peña, S. Díez-Muñiz, C. Nombela, C. L. Peterson, J. Arroyo (2012). Mol Biol Cell 23:2805-2817. <https://doi.org/10.1091/mbc.E12-04-0278>.

-A. Köhler, P. Pascual-García, A. Llopis, M. Zapater, F. Posas, E. Hurt, S. Rodriguez-Navarro (2006). Mol Biol Cell 17:4228-4236. <https://doi.org/10.1091/mbc.E06-02-0098>.

-A. B. Sanz, R. García, J. M. Rodríguez-Peña, C. Nombela, J. Arroyo (2016). Nucleic Acids Res 44:7159-7172. https://doi.org/10.1093/nar/gkw324.

-S. D. Turner, A. R. Ricci, H. Petropoulos, J. Genereaux, I. S. Skerjanc, C. J. Brandl (2002) Mol Cell Biol 22:4011-4019. https://doi.org/10.1128/MCB.22.12.4011-4019.2002.
