# Supplementary material for: Sus1 Modulates Chromatin Remodeling and Gene Expression via the Cell Wall Integrity Pathway in Saccharomyces cerevisiae
Source: FASEB J. 2026 Apr 28;40:e71848. doi: 10.1096/fj.202504656RR (PMC13123632; doi:10.1096/fj.202504656RR)
Supplement: Supplementary file 3 — Table S2: Primers used in this work. [file FSB2-40-e71848-s003.docx]

**Table S2: Primers used in this work.**

| Primer name | Sequence | Position | Efficiency |
| --- | --- | --- | --- |
| ***KDX1*** |  |  |  |
| KDX1-BOX1 UP | AATTTCACTGTTTCCGTTAACTGTTG | -453 | 98.3 |
| KDX1-BOX1 DW | TCCTACGGTGCTTCCTGCAT | -313 |  |
| KDX1-POL II UP | GCAAAAAGCCTATTTAATGTAAGTCC | -143 | 95.1 |
| KDX1-POL II DW | AGGATAAAATCTTGGCCGAATG | 56 |  |
| KDX1-ORF2 UP | CGACGGCTTCATTAAGGGTTATA | 555 | 101.9 |
| KDX1-ORF2 DW | CCTACCAAGTAGTTCGGCCAAG | 687 |  |
| KDX1-ORF3 UP | TCCTATGTAAGCCAACTTCCATCA | 1210 | 92.1 |
| KDX1-ORF3 DW | TCCGTTCAGTAGTTCACTATTTTCATG | 1359 |  |
| ***YPL088W*** |  |  |  |
| YPL088W-BOX UP | ACGTTTCTTTTTGTCTGTCCTTT | -573 | 96.3 |
| YPL088W-BOX DW | CGGGTGATGAATGCCAAGC | -423 |  |
| YPL088W-POL II UP | ATTAGGACCCAGAGAACAAGGT | -91 | 109.3 |
| YPL088W-POL II DW | TCGGTGATATCTTAAGACCTGAGT | 55 |  |
| YPL088W-ORF2 UP | CTGCAGTTCACAGCCGATAA | 562 | 105.2 |
| YPL088W-ORF2 DW | CTCGTGCGTTAGGAGACCAT | 712 |  |
| YPL088W-ORF3 UP | TGGGATTGAACACTACAGCAAGA | 911 | 93.0 |
| YPL088W-ORF3 DW | CCTTCTATATATCAGTGGGCCCATG | 1064 |  |
| ***PRM5*** |  |  |  |
| PRM5-BOX UP | TTCTTGCTTTCTCTTTTTGGCT | -287 | 98.9 |
| PRM5-BOX DW | GCGTTCCTTTGAATGATCTCA | -106 |  |
| PRM5-POL II UP | CATCGGTGCAAAGAAACAGTTA | -106 | 93.7 |
| PRM5-POL II DW | AATTTTGGAAGGCCTCTCTTG | 41 |  |
| PRM5-ORF2 UP | TATGGATTATCCCGCCAAAG | 393 | 91.9 |
| PRM5-ORF2 DW | AGCGGCCTTTCGAAATTACT | 545 |  |
| PRM5-ORF3 UP | CCAAGTCGAAAGCTGCTTAAT | 805 | 92.3 |
| PRM5-ORF3 DW | ATCCTGTTCTTTCCCCTCCA | 951 |  |
| ***CROV*** |  |  |  |
| CROV UP | GGGAAGACCAATTTCATGACGA | intergenic region of chromosome V | 95.6 |
| CROV DW | AATCTGCGTAAAAATGGCGTAAA |  |  |

**ChIP** (locations are indicated by the distance from the respective ATG initiation codon)

| Gene | Primer name | Sequence | Efficiency |
| --- | --- | --- | --- |
| *KDX1* | YKL161C-UP | TGAATTATCAAGAATGCACAAAAGC | 94.9 |
|  | YKL161C-DW | TCCTTCCCTTCAAACATTGGTT |  |
| *YPL088W* | YPL088W-UP | ATGGCTTCCTAACGCACGAG | 102.4 |
|  | YPL088W-DW | GTCCTTCGACACCTTTTCCA |  |
| *PIR3* | YKL163W-UP | TCGTTGCCAACAGACAGTTC | 99.9 |
|  | YKL163W-DW | TTCATGGCACTGAGAACCAA |  |
| *CRG1* | YHR209W-UP | TCGATTTGGAGATATTGAAGTCACA | 99.3 |
|  | YHR209W-DW | GCATTTGGGTCCGAAGGA |  |
| *PRM5* | YIL117C-UP | AGACATAAGGAAACCCGCAAAAA | 109.9 |
|  | YIL117C-DW | ACGATTTACGCTACCATCACTTTCT |  |
| *CWP1* | YKL096W-UP | CCTCTGCTAGCTCCCCAACTG | 97.7 |
|  | YKL096W-DW | TGTGTTTGGAGCTTGGATTTGT |  |
| *SLT2* | YHR030C-UP | AAGGCATGATGCAGATTTCC | 97.5 |
|  | YHR030C-DW | AAGCGTGCCGTTATCATTCT |  |
| *RLM1* | YPL089C-UP | CCGCATATAATGGAAATACCG | 101.1 |
|  | YPL089C-DW | TCTCCTGAAATATCAGTCGAAAAA |  |
| *ACT1* | ACT-UP | ACGAAAGATTCAGAGCCCCA | 107.4 |
|  | ACT-DW | GCAGATTCCAAACCCAAAACA |  |

**RT-qPCR**

**MNase** (locations are indicated by the distance from the respective ATG initiation codon)

**Strain generation** (INT for interruptions; TAG for epitope tagging and K for verification)

| **Primer name** | **Sequence** |
| --- | --- |
| *SUS1*-INT-1 | TGCGACAAAATCAGAAGTAACAATTCTGGCCTTCACTCCACGTACGCTGCAGGTCGAC |
| *SUS1*-INT-2 | ATGTAATAATATTGGGAATTAAGGTGCATTTTCGTATCCTATCGATGAATTCGAGCTCG |
| *SUS1*-TAG-1 | GCATATGTAATAATATTGGGAATTAAGGTGCATTTTCGTATCCGAATTCGAGCTCGTTTAAAC |
| *SUS1*-TAG-2 | GCAAATAAGGGAATTTCTTGAAGAGATTGTAGATACACAACGGATCCCCGGGTTAATTAA |
| *SUS1*-K2 | GGCGAAACTGCTTTTGTAGC |
| *SUS1*-K3 | GGAAACAGTGAAAGAGGAGG |
| *SUS1*-TAG-K2 | CGAACTAAAAGCCAGACTAC |
| *UBP8*-INT-1 | CGTCCTACTTGAAACCCTGCTTTTTTTATTTGTTATTAA |
| *UBP8*-INT-2 | CTTCTTTTTTGTTTTATTATTATTGTTGAATGCTATTTG |
| *UBP8*-K2 | GGGAAATTCATTGGGTGCTG |
| *UBP8*-K3 | CAGAAGGGGAAAAGGAAGAG |
| *RLM1*-TAG-1 | TCGACTGATATTTCAGGAGAAAAAAATTCAAGCAAAATAGAATTCGAGCTCGTTTAAAC |
| *RLM1*-TAG-2 | CTTATGCTTGGAATATTCATACTGGTCAAATTTTTTGGTCGGATCCCCGGGTTAATTAA |
| *RLM1*-K3 | AATTACGCCAATCTTCCAAGTG |
| *RLM1-*TAG-K2 | GTGACTCGAACAATCAGTC |
| *SAC3-*TAG-1 | ATAGAAAAAATGCACATTTCTTTTGTTTATATATTACAAATGCTGAATTCGAGCTCGTTTAAAC |
| *SAC3*-TAG-2 | GATCTTGATCGATTCTGTCAAGAAGAAAGTAAATAATGATCGGATCCCCGGGTTAATTAA |
| *SAC3*-K2 | GCCGAAATCTTACTAGCAAG |
| *SAC3*-K3 | GCCAGTGTCTATACTACCTG |
| *SAC3-*TAG-K2 | CCAGCTGCAAGTTTTAGCGG |

| Primer name | Sequence | Position | Efficiency |
| --- | --- | --- | --- |
| *KDX1* tiling 1-F  *KDX1* tiling 1-R | TACTCCATCGCCATCCCTTTT  AATGTCGATACTGTGTTACGTTTGC | -699  -602 | 97.8 |
| *KDX1* tiling 2-F  *KDX1* tiling 2-R | CTTGCAAACGTAACACAGTATCGA  GACGTCTTGTGGGCTGAAAAC | -629  -530 | 95.1 |
| *KDX1* tiling 3-F  *KDX1* tiling 3-R | CCGTAGTTTTCAGCCCACAAG  ATTGGGGACTTTCAAGTAACGAA | -555  -455 | 98.8 |
| *KDX1* tiling 4-F  *KDX1* tiling 4-R | ATTCGTTACTTGAAAGTCCCCAAT  GTGTTTCTCCGGTGTTGTTCAA | -478  -377 | 97.2 |
| *KDX1* tiling 5-F  *KDX1* tiling 5-R | GATTATTTTGAACAACACCGGAGA  AATTCCTACGGTGCTTCCTGC | -405  -310 | 97.9 |
| *KDX1* tiling 6-F  *KDX1* tiling 6-R | TCAATCAATGCAGGAAGCACC  TTTGTGGCAGCGCTTTTTACT | -339  -236 | 97.4 |
| *KDX1* tiling 7-F  *KDX1* tiling 7-R | AGTAAAAAGCGCTGCCACAAA  AAAAGTACCTTCTTTTGTTTCTTCCAA | -256  -159 | 95.7 |
| *KDX1* tiling 8-F  *KDX1* tiling 8-R | TTGGAAGAAACAAAAGAAGGTACTTT  CCATTTTGTCCCTTTTCATCCA | -185  -79 | 95.1 |
| *KDX1* tiling 9-F  *KDX1* tiling 9-R | GAGACTGGATGAAAAGGGACAAA  CGGTGTCAGTCGCCATTTAAT | -105  +15 | 97.8 |
| *KDX1* tiling 10-F  *KDX1* tiling 10-R | TCACAATTAAATGGCGACTGACA  CCCCGACCAATCTTACCTGTC | -10  +94 | 99.9 |
| *KDX1* tiling 11-F  *KDX1* tiling 11-R | CATTTTCATTTGACAGGTAAGATTGG  TCTGATAGCCACGTGAGTTTCCT | +63  +161 | 95.8 |

All primer pairs used in qPCR analysis were validated using standard and melting curve analyses by serial dilutions of genomic DNA from the wild-type strain BY4741, showing a single peak in dissociation curves and amplification efficiencies ranging from 90% to 110%.
