# Supplementary material for: Sus1 Modulates Chromatin Remodeling and Gene Expression via the Cell Wall Integrity Pathway in Saccharomyces cerevisiae
Source: FASEB J. 2026 Apr 28;40:e71848. doi: 10.1096/fj.202504656RR (PMC13123632; doi:10.1096/fj.202504656RR)
Supplement: Supplementary file 4 — Table S3: List of genes induced and repressed under CR treatment (30 μg/mL, 3 h) in a WT strain and its dependence on Sus1 and Gcn5. [file FSB2-40-e71848-s004.docx]

**Table S3. List of genes induced and repressed under CR treatment (30 μg/mL, 3h) in a WT strain and its dependence on Sus1 and Gcn5.**

| ORF | Gene Symbol | WT CR /WT | *sus1*Δ CR /*sus1*Δ | *sus1*Δ /WT | Dependence on Sus1 | Dependence on Gcn5 |
| --- | --- | --- | --- | --- | --- | --- |
| *YNR001C* | *CIT1* | 2.12 | 1.29 | 1.17 | ● | - |
| *YPL052W* | *OAZ1* | 2.08 | 1.34 | 1.15 | ● | - |
| *YBR182C* | *SMP1* | 2.33 | 1.41 | 1.61 | ● | - |
| *YFL030W* | *AGX1* | 2.01 | 1.08 | 1.63 | ● | - |
| *YDR380W* | *ARO10* | 2.06 | 1.37 | 0.69 | ● | - |
| *YOL122C* | *SMF1* | 2.06 | 1.38 | 0.82 | ● | - |
| *YIL122W* | *POG1* | 2.09 | 1.40 | 1.24 | ● | - |
| *YJL099W* | *CHS6* | 2.04 | 1.41 | 1.02 | ● | - |
| *YBL078C* | *ATG8* | 2.20 | 1.46 | 1.12 | ● | - |
| *YNL293W* | *MSB3* | 2.01 | 1.48 | 1.00 | ● | - |
| *YLR121C* | *YPS3* | 6.49 | 4.16 | 0.83 | ● | □ |
| *YDR055W* | *PST1* | 3.60 | 2.19 | 1.51 | ● | □ |
| *YOL159C* | *YOL159C* | 2.88 | 1.85 | 0.98 | ● | □ |
| *YKL107W* | *YKL107W* | 2.27 | 1.41 | 0.93 | ● | □ |
| *YCR073C* | *SSK22* | 2.92 | 1.59 | 1.72 | ● | □ |
| *YNL192W* | *CHS1* | 2.24 | 1.64 | 1.14 | ● | □ |
| *YPL110C* | *GDE1* | 2.26 | 1.43 | 1.20 | ● | □ |
| *YGL156W* | *AMS1* | 2.39 | 1.55 | 1.35 | ● | □ |
| *YMR238W* | *DFG5* | 2.46 | 1.55 | 1.06 | ● | □ |
| *YBR071W* | *YBR071W* | 2.09 | 1.58 | 0.83 | ● | □ |
| *YKL163W* | *PIR3* | 43.94 | 8.95 | 2.19 | ● | ■ |
| *YKL161C* | *KDX1* | 17.66 | 9.12 | 1.63 | ● | ■ |
| *YHR209W* | *CRG1* | 11.28 | 4.89 | 1.29 | ● | ■ |
| *YDR034W-B* | *YDR034W-B* | 12.07 | 3.03 | 1.69 | ● | ■ |
| *YBR056W-A* | *YBR056W-A* | 6.45 | 3.52 | 1.28 | ● | ■ |
| *YPL088W* | *YPL088W* | 7.49 | 3.54 | 1.12 | ● | ■ |
| *YGR032W* | *GSC2* | 5.88 | 2.35 | 1.26 | ● | ■ |
| *YOR134W* | *BAG7* | 6.48 | 3.43 | 1.48 | ● | ■ |
| *YNR065C* | *YNR065C* | 5.10 | 2.24 | 1.08 | ● | ■ |
| *YJL108C* | *PRM10* | 4.93 | 2.81 | 1.06 | ● | ■ |
| *YPR005C* | *HAL1* | 4.12 | 2.28 | 1.04 | ● | ■ |
| *YGR213C* | *RTA1* | 4.55 | 2.21 | 2.80 | ● | ■ |
| *YMR316W* | *DIA1* | 3.33 | 2.11 | 1.62 | ● | ■ |
| *YLR414C* | *PUN1* | 2.97 | 1.83 | 1.27 | ● | ■ |
| *YJL161W* | *FMP33* | 3.16 | 1.82 | 1.03 | ● | ■ |
| *YIR039C* | *YPS6* | 3.32 | 1.56 | 1.29 | ● | ■ |
| *YHL022C* | *SPO11* | 4.70 | 1.78 | 1.66 | ● | ■ |
| *YJL155C* | *FBP26* | 2.61 | 1.66 | 1.29 | ● | ■ |
| *YLR267W* | *BOP2* | 2.48 | 1.19 | 1.05 | ● | ■ |
| *YOR306C* | *MCH5* | 2.99 | 1.84 | 1.43 | ● | ■ |
| *YPR078C* | *YPR078C* | 3.29 | 2.04 | 1.05 | ● | ■ |
| *YLR040C* | *AFB1* | 2.48 | 1.35 | 0.62 | ● | ■ |
| *YDR018C* | *YDR018C* | 2.46 | 1.46 | 1.01 | ● | ■ |
| *YMR118C* | *SHH3* | 3.51 | 1.34 | 1.73 | ● | ■ |
| *YKR046C* | *PET10* | 2.38 | 1.54 | 0.76 | ● | ■ |
| *YBR295W* | *PCA1* | 2.55 | 1.50 | 1.24 | ● | ■ |
| *YIL108W* | *YIL108W* | 2.54 | 1.59 | 1.19 | ● | ■ |
| *YPR194C* | *OPT2* | 2.60 | 1.53 | 1.42 | ● | ■ |
| *YGR161C* | *RTS3* | 2.24 | 1.43 | 1.26 | ● | ■ |
| *YGR166W* | *TRS65* | 2.01 | 1.54 | 1.05 | ● | ■ |
| *YNL294C* | *RIM21* | 2.37 | 1.49 | 0.99 | ● | ■ |
| *YEL060C* | *PRB1* | 2.80 | 1.42 | 1.54 | ● | ■ |
| *YMR315W-A* | *YMR315W-A* | 2.21 | 1.47 | 1.10 | ● | ■ |
| *YGR097W* | *ASK10* | 2.26 | 1.64 | 1.02 | ● | ■ |
| *YGL230C* | *YGL230C* | 2.22 | 1.36 | 1.56 | ● | ■ |
| *YIR028W* | *DAL4* | 2.66 | 1.23 | 1.64 | ● | ■ |
| *YBR005W* | *RCR1* | 2.06 | 1.49 | 1.21 | ● | ■ |
| *YDR001C* | *NTH1* | 2.16 | 1.42 | 1.08 | ● | ■ |
| *YMR008C* | *PLB1* | 2.30 | 1.29 | 1.21 | ● | ■ |
| *YOR220W* | *RCN2* | 2.15 | 1.49 | 1.13 | ● | ■ |
| *YDR261C* | *EXG2* | 2.18 | 1.70 | 1.22 | ○ | ■ |
| *YNL053W* | *MSG5* | 2.51 | 1.85 | 0.82 | ○ | ■ |
| *YEL058W* | *PCM1* | 2.37 | 1.74 | 1.07 | ○ | ■ |
| *YPL089C* | *RLM1* | 2.36 | 1.98 | 1.16 | ○ | ■ |
| *YLR120C* | *YPS1* | 2.35 | 2.11 | 0.97 | ○ | ■ |
| *YIL023C* | *YKE4* | 2.38 | 2.19 | 1.04 | ○ | ■ |
| *YPL221W* | *FLC1* | 2.65 | 1.86 | 0.98 | ○ | ■ |
| *YAL053W* | *FLC2* | 2.94 | 2.34 | 0.92 | ○ | ■ |
| *YHR030C* | *SLT2* | 3.74 | 2.71 | 1.03 | ○ | ■ |
| *YMR104C* | *YPK2* | 3.63 | 2.44 | 1.18 | ○ | ■ |
| *YKR061W* | *KTR2* | 4.03 | 2.64 | 1.00 | ○ | ■ |
| *YOR208W* | *PTP2* | 2.76 | 1.98 | 0.97 | ○ | □ |
| *YPL067C* | *YPL067C* | 2.73 | 2.06 | 1.16 | ○ | □ |
| *YKL104C* | *GFA1* | 2.73 | 2.21 | 1.11 | ○ | □ |
| *YGR189C* | *CRH1* | 2.18 | 2.33 | 0.97 | ○ | □ |
| *YJL107C* | *YJL107C* | 2.83 | 2.40 | 1.81 | ○ | □ |
| *YDR077W* | *SED1* | 2.05 | 2.42 | 0.72 | ○ | - |
| *YNL058C* | *YNL058C* | 2.41 | 2.63 | 0.85 | ○ | □ |
| *YDR085C* | *AFR1* | 4.46 | 3.31 | 0.97 | ○ | □ |
| *YKL096W* | *CWP1* | 2.54 | 3.41 | 0.56 | ○ | □ |
| *YIL117C* | *PRM5* | 4.18 | 4.50 | 0.70 | ○ | □ |
| *YLR194C* | *YLR194C* | 6.39 | 4.70 | 1.09 | ○ | □ |
| *YKR091W* | *SRL3* | 6.05 | 5.94 | 0.70 | ○ | □ |
| *YLR031W* | *YLR031W* | 2.69 | 1.20 | 1.83 | ◇ | - |
| *YGR110W* | *CLD1* | 2.14 | 1.42 | 1.69 | ◇ | - |
| *YKR053C* | *YSR3* | 2.18 | 1.56 | 1.73 | ◇ | ■ |
| *YCR100C* | *YCR100C* | 2.33 | 1.23 | 1.93 | ◇ | ■ |
| *YNR064C* | *YNR064C* | 2.11 | 1.09 | 1.87 | ◇ | ■ |
| *YOR137C* | *SIA1* | 2.04 | 1.23 | 1.82 | ◇ | - |
| *YJL116C* | *NCA3* | 2.33 | 1.05 | 2.27 | ◇ | - |
| *YIL072W* | *HOP1* | 2.14 | 1.08 | 2.29 | ◇ | - |
| *YGR271C-A* | *EFG1* | 0.50  0.50  0.50 | 1.00 1.00  1.00  1.00 | 0.51 0.51 | ● | - |
| *YKR083C* | *DAD2* | 0.50 | 0.87 0.87 | 0.71 0.71 | ● | - |
| *YPL189W* | *GUP2* | 0.42 0.42 | 0.59 0.59 | 2.43 2.43 | ○ | ∇ |
| *YDL182W* | *LYS20* | 0.50 0.50 | 0.72 0.72 | 1.13 1.13 | ○ | - |
| *YMR230W-A* | *YMR230W-A* | 0.49 0.49 | 0.72 | 0.67 | ○ | □ |
| *YIL019W* | *FAF1* | 0.50 | 0.74 0.74 | 1.25 1.25 | ○ | ∇ |
| *YAL059W* | *ECM1* | 0.50 0.50 | 0.79 0.79 | 1.10 1.10 | ◇ | - |
| *YAR068W* | *YAR068W* | 0.49 0.49 | 0.97 | 1.12 1.12 | ◇ | - |
| *YJL212C* | *OPT1* | 0.34 0.34 | 0.69 0.69 | 0.79 0.79 | ◇ | □ |
| *YKL103C* | *APE1* | 0.31 0.31 | 0.66 0.66 | 0.78 0.78 | ◇ | ∇ |
| *YKL029C* | *MAE1* | 0.49 0.49 | 0.77 0.77 | 1.26 1.26 | ◇ | - |
| *YDR078C* | *SHU2* | 0.37 0.37 | 0.62 0.62 | 1.22 1.22 | ◇ | ■ |
| *YBL101W-A* | *YBL101W-A* | 0.25 0.25 | 0.91 0.91 | 3.65 3.65 | ◇ | ∇ |

The list includes ORFs whose transcripts are either induced (gene expression ratio WT CR/WT ≥ 2, shown in yellow) or repressed (expression ratio WT CR/WT ≤ 0.5, shown in blue) after CR treatment (30 μg/mL, 3 hours) in the WT strain. It also includes the gene expression ratios under CR treatment for the *sus1*Δ mutant (*sus1*Δ CR/*sus1*Δ) and the basal expression ratios of the *sus1*Δ mutant vs the WT strain under non-stress conditions (*sus1*Δ/WT). Genes were classified as Sus1-dependent and/or Gcn5-dependent, based on the data analysis described in Materials and Methods, using the following categories: Sus1-dependent genes (●); Sus1-independent genes (○); basal Sus1-dependent genes (◇); Gcn5-dependent genes (■); Gcn5-independent genes (□); basal Gcn5-dependent genes (∇); and genes with no data are available (-). Gcn5 dependence was evaluated using microarray data from the ***gcn5*Δ** mutant, previously obtained by our research group (Sanz et al., 2016).
